# Undiagnosed Cancer Cases in the US During the First 10 Months of the COVID-19 Pandemic

**DOI:** 10.1001/jamaoncol.2023.6969

**Published:** 2024-02-22

**Authors:** Todd Burus, Feitong Lei, Bin Huang, W. Jay Christian, Pamela C. Hull, Amanda R. Ellis, Svetla Slavova, Thomas C. Tucker, Krystle A. Lang Kuhs

**Affiliations:** 1 Markey Cancer Center, University of Kentucky, Lexington; 2 Department of Biostatistics, College of Public Health, University of Kentucky, Lexington; 3 Division of Cancer Biostatistics, College of Medicine, University of Kentucky, Lexington; 4 Kentucky Cancer Registry, Markey Cancer Center, University of Kentucky, Lexington; 5 Department of Epidemiology & Environmental Health, College of Public Health, University of Kentucky, Lexington; 6 Department of Behavioral Science, College of Medicine, University of Kentucky, Lexington; 7 Kentucky Injury Prevention & Research Center, University of Kentucky, Lexington

## Abstract

**Importance:**

The COVID-19 pandemic disrupted the normal course of cancer screening and detection in the US. A nationwide analysis of the extent of this disruption using cancer registry data has not been conducted.

**Objective:**

To assess the observed and expected cancer rate trends for March through December 2020 using data from all 50 US states and the District of Columbia.

**Design, Settings, and Participants:**

This was a population-based cross-sectional analysis of cancer incidence trends using data on cases of invasive cancer diagnosis reported to the US Cancer Statistics from January 1, 2018, through December 31, 2020. Data analyses were performed from July 6 to 28, 2023.

**Exposure(s):**

Age, sex, race, urbanicity, and state-level response to the COVID-19 pandemic at the time of cancer diagnosis.

**Main Outcomes and Measures:**

Used time-series forecasting methods to calculate expected cancer incidence rates for March 1 through December 31, 2020, from prepandemic trends (January 2018-February 2020). Measured relative difference between observed and expected cancer incidence rates and numbers of potentially missed cancer cases.

**Results:**

This study included 1 297 874 cancer cases reported in the US from March 1 through December 31, 2020, with an age-adjusted incidence rate of 326.5 cases per 100 000 population. Of the observed cases, 657 743 (50.7%) occurred in male patients, 757 106 (58.3%) in persons 65 years or older, and 1 066 566 (82.2%) in White individuals. Observed rates of all-sites cancer incidence in the US were 28.6% (95% prediction interval [PI], 25.4%-31.7%) lower than expected during the height of the COVID-19 pandemic response (March-May 2020); 6.3% (95% PI, 3.8%-8.8%) lower in June to December 2020; and overall, 13.0% (95% PI, 11.2%-14.9%) lower during the first 10 months of the pandemic. These differences indicate that there were potentially 134 395 (95% PI, 112 544-156 680) undiagnosed cancers during that time frame. Prostate cancer accounted for the largest number of potentially missed cases (22 950), followed by female breast (16 870) and lung (16 333) cancers. Screenable cancers saw a total rate reduction of 13.9% (95% PI, 12.2%-15.6%) compared with the expected rate. The rate of female breast cancer showed evidence of recovery to previous trends after the first 3 months of the pandemic, but levels remained low for colorectal, cervical, and lung cancers. From March to May 2020, states with more restrictive COVID-19 responses had significantly greater disruptions, yet by December 2020, these differences were nonsignificant for all sites except lung, kidney, and pancreatic cancer.

**Conclusions and Relevance:**

This cross-sectional analysis of cancer incidence trends found a substantial disruption to cancer diagnoses in the US during the first 10 months of the COVID-19 pandemic. The overall and differential findings can be used to inform where the US health care system should be looking to make up ground in cancer screening and detection.

## Key Points

**Question:**

What association did the COVID-19 pandemic have with cancer diagnoses in the US from March 1 through December 31, 2020?

**Findings:**

This population-based cross-sectional study found that all-sites cancer incidence in the US was significantly lower than expected in March through December 2020, with 134 395 potentially undiagnosed cancer cases.

**Meaning:**

These findings identify a substantial deficit of diagnosed cancer cases in the US during the COVID-19 pandemic in 2020, which underscores the need to reengage individuals in recommended cancer screenings and routine health care visits.

## Teaser

This cross-sectional analysis evaluates observed and expected cancer rate trends in the US to quantify the potentially undetected cancer cases associated with the COVID-19 pandemic response.

## Introduction

The discovery of the SARS-CoV-2 virus in 2019 and the ensuing global COVID-19 pandemic led to unprecedented disruptions across all parts of society. Yet while the world focused on the threat posed by a novel respiratory virus, the threat of cancer remained ever present, and any associated reduction in observed cancer incidence during this period is of particular concern. Unlike other population health outcomes studied, a decline in new cancer diagnoses in 2020 does not indicate that cancer occurrence in the US decreased, but rather that new cancers were undetected.^1,2,3^ The longer cancer exists undetected, the greater the risk of tumor progression and the lower the chances of survival and other positive outcomes for patients.^4^

The negative association of the pandemic response with cancer diagnoses was widely anticipated. However, the data needed to assess the extent of this relationship were not available in the US until recently.^5,6,7^ The present study uses 2020 US cancer incidence data to conduct an in-depth analysis of disruptions and delays in cancer diagnoses during the first year of the COVID-19 pandemic.

## Methods

 This cross-sectional analysis was deemed exempt from review by the University of Kentucky Institutional Review Board. Informed consent was waived because the study used only publicly available deidentified data. We followed the Strengthening the Reporting of Observational Studies in Epidemiology (STROBE) reporting guidelines.

### Data Sources

This study used cancer incidence data from the US Cancer Statistics 2001-2020 Public Use Database, which contains submissions from the National Program of Cancer Registries (US Centers for Disease Control and Prevention) and the SEER (Surveillance, Epidemiology, and End Results) program (US National Cancer Institute), and covers approximately 99.7% of cancer cases in all 50 states and the District of Columbia.^8^ Invasive cancer incidence rates were calculated based on month of diagnosis for January 1, 2018, through December 31, 2020, and age-adjusted to the 2000 US Standard Population using SEER*Stat statistical software, version 8.4.1 (US National Cancer Institute). In addition to incidence rates, patient demographic information (age, sex, race, urbanicity, and state of residence at time of diagnosis) and cancer stage at diagnosis were also extracted. Indiana and Nevada were excluded due to the unavailability of 2020 data.

### Study Design

In this population-based cross-sectional study, we identified patients with any invasive cancer diagnosis between 2018 and 2020 to calculate monthly all-sites cancer incidence rates. Cases with an unknown month of diagnosis were excluded from trend analysis (<0.5% of cases annually) (eFigure 1 in Supplement 1). Additional cancer sites and site groupings were identified according to the *International Classification of Diseases for Oncology, Third Revision (ICD-O-3), World Health Organization, 2008.*^9^

We identified patients as having a screenable cancer in accordance with US Preventive Services Task Force high-grade recommendations: (1) lung and bronchus; (2) colon and rectum; (3) breast (females only); and (4) cervix uteri. We calculated individual rates for each of these 4 screenable cancers along with 7 cancer sites on the US Cancer Statistics’ list of top 10 sites for cancer incidence in 2020^10^: prostate; corpus and uterus, not otherwise specified; melanoma of the skin; urinary bladder; non-Hodgkin lymphoma; kidney and renal pelvis; and pancreas. Incidence rates were calculated among the total eligible population and by various patient characteristics (age, sex, race, urbanicity, state of residence, and state-level response to COVID-19) or cancer stage at diagnosis. Age at diagnosis was grouped by Medicare eligibility as younger than 65 years or 65 years and older. Race was classified according to the Race Recode variable, with rates captured for only Black or White patients due to data availability; ethnicity was not captured. Urbanicity was based on 2013 Rural-Urban Continuum Codes, with codes 1 to 3 defined as metropolitan and codes 4 to 9 defined as nonmetropolitan.^11^ The COVID-19 response of the state of residence was classified according to the length of state-level stay-at-home orders during spring 2020 (precise dates for each state are available in the eMethods in Supplement 1). A state was defined as less restrictive if its stay-at-home order had a duration of 42 days or less, and more restrictive if the duration was greater than 42 days. Forty-two days represents the difference in median start and end dates of state-level stay-at-home orders and created no ambiguity in classification (eMethods in Supplement 1).^12^ Cancer stage at diagnosis was classified according to Merged Summary Stage (SEER*Stat statistical software, version 8.4.1). Early stage was defined as localized disease, and late stage included any regional or distant metastasis.

### Statistical Analysis

Monthly age-adjusted cancer incidence rates measured from January 2018 through December 2020 were converted to time series for each cancer grouping and cancer site considered. Time series were fit to (potentially seasonal) autoregressive integrated moving average (ARIMA) models to perform interrupted time-series analysis according to the methods described by Schaffer et al.^13^ ARIMA models can combine nonseasonal and seasonal differencing with autoregressive and moving average models and are well suited to handling time-series data that include seasonal trends and serial autocorrelation. Standard ARIMA models are defined by the autoregressive model order (*p*), the order of differencing (*d*), and the moving average model order (*q*). A seasonal component may be added with corresponding parameters *P, D*, *Q,* and a value *m* for the seasonal period. The general form for the final model is ARIMA(*p,d,q*)(*P,D,Q*)*_m_*.

We postulated and included 2 potential roles of the COVID-19 pandemic in 2020 as exogenous regressors in our model-building process.^13,14^ First, we proposed a pulse effect covering the early-pandemic period of March 1 to May 31, 2020. A pulse effect occurs when an interruption causes a sudden increase or decrease in the outcome of interest, followed by a rapid return to previous trends. The inclusion of this effect captured the average rate reduction during the period of widespread stay-at-home orders. The second postulated effect was a step change for the later pandemic period from June 1 to December 31, 2020. A step-change effect is an interruption that involves a sudden and sustained change in baseline rates from the preinterruption trend. This effect helped to assess whether rates returned to previous trends after the period of stay-at-home orders.

We performed log transformations of the age-adjusted rates to stabilize variance before fitting models. Potential models were then fit iteratively using the effects described, with the best possible model chosen according to minimum Akaike information criteria.^13^ After fitting a best model, model coefficients were assessed and the Ljung-Box test was performed to check for residual autocorrelation. If the model coefficient for the step-change effect was not significant or the Ljung-Box test detected residual autocorrelation, the model was refit without the step-change regressor. This process was repeated to test for significant pulse effect. Final model parameters were recorded and used to forecast counterfactual monthly rates, with pointwise 95% prediction intervals (PIs) assuming the absence of the COVID-19 pandemic. We constructed expected rates for the aggregate periods of March to May 2020, June to December 2020, and March to December 2020, using forecast simulations with 10 000 repetitions (additional details are available in the eMethods in Supplement 1). We calculated absolute and relative differences of observed from expected rates for each period. The relative difference of observed rates from expected rates was calculated as: (observed rate minus expected rate) divided by expected rate. We estimated potentially missed cases for population cancer rates by multiplying absolute differences by the appropriate standard population (or standard population divided by 2 for the 4 sex-specific cancer sites). In total, we fit and analyzed 174 different ARIMA models.

Statistical significance using formal tests was assessed at a level of *P* < .05, and all hypotheses were 2-sided. Observed rates were deemed significantly different from expected rates when they were not within the 95% PI (for which there is no formal significance test, and thus no *P* values to report). All analyses were conducted from July 6 to 28, 2023, using R statistical software, version 4.2.3 (The R Foundation for Statistical Computing).

## Results

### Population Cancer Rates

This study included 1 297 874 cancer cases reported in the US from March 1 through December 31, 2020, with an age-adjusted incidence rate of 326.5 cases per 100 000 population. Of the observed cases, 657 743 (50.7%) occurred in male patients, 757 106 (58.3%) in persons 65 years or older, and 1 066 566 (82.2%) in White individuals (Table 1).

**Table 1.** Comparison of All-Sites Cancer Cases Before and During the COVID-19 Pandemic, USCS Public Use Database^8^

Characteristic	No. (%)	
	Before COVID-19 pandemic^a^	During COVID-19 pandemic^b^
Total cases	3 800 931	1 302 313
Included cases^c^	3 787 834 (99.7)	1 297 874 (99.7)
Cases missing month^d^	13 097 (0.3)	4439 (0.3)
Sex		
Female	1 859 253 (49.1)	640 131 (49.3)
Male	1 928 581 (50.9)	657 743 (50.7)
Age, y		
<65	1 604 318 (42.4)	540 768 (41.7)
≥65	2 183 516 (57.6)	757 106 (58.3)
Race		
Black	425 189 (11.2)	144 743 (11.2)
White	3 132 990 (82.7)	1 066 556 (82.2)
Other^e^	229 655 (6.1)	86 575 (6.7)
Urbanicity		
Metropolitan	3 094 333 (81.7)	1 059 222 (81.6)
Nonmetropolitan	599 370 (15.8)	205 297 (15.8)
Unknown	94 131 (2.5)	33 355 (2.6)
Cancer stage		
Early	1 697 184 (44.8)	559 810 (43.1)
Late	1 663 958 (43.9)	590 829 (45.5)
Unknown	426 692 (11.3)	147 235 (11.3)
State-level COVID-19 response		
More restrictive	2 204 667 (58.2)	751 374 (57.9)
Less restrictive	1 583 167 (41.8)	546 500 (42.1)

Abbreviation: USCS, US Cancer Statistics.

^a^ From January 1, 2018, through February 29, 2020; these cases were used to build autoregressive integrated moving average models.

^b^ From March 1 through December 31, 2020.

^c^ Denominator used for calculating subgroup percentages.

^d^ Cases missing month of cancer diagnosis in 2020 were assigned to the pandemic study period.

^e^ Included American Indian, Alaska Native, Asian, Pacific Islander, and unknown.

**Figure 1.**
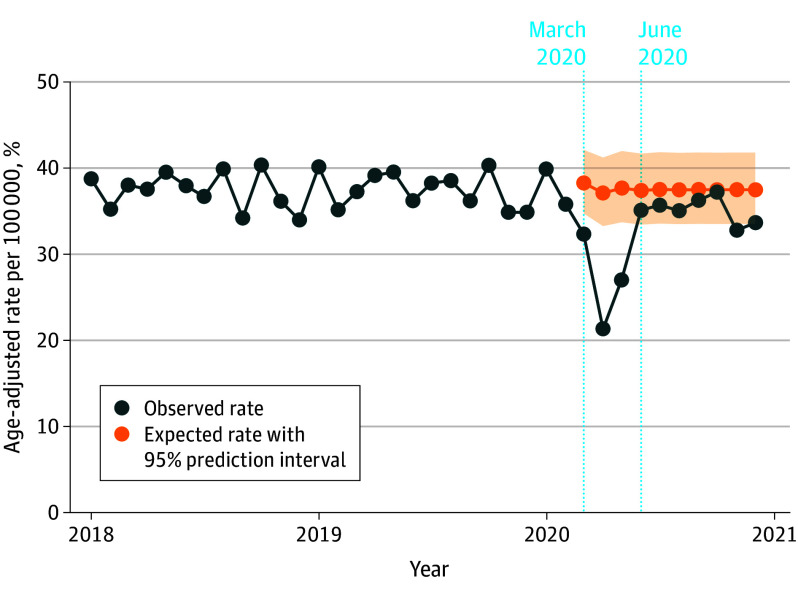
All-Sites Cancer Incidence Rates in the US for January 1, 2018, Through December 31, 2020 Monthly observed all-sites cancer incidence rates. Expected rates in the absence of the COVID-19 pandemic displayed starting in March 2020. The beginnings of the 2 pandemic periods considered (March-May 2020 and June-December 2020) are noted.

Selected models suggest that age-adjusted all-sites cancer incidence rates followed an ARIMA (1,0,0) model between January 1, 2018, and December 31, 2020 (Figure 1). During the widespread stay-at-home orders in March to May 2020, overall cancer diagnoses fell by a significant 28.6% (95% PI, 25.4%-31.7%) (Table 2). Rates recovered slightly from June to December 2020; yet a significant step-change effect was still present, corresponding to a 6.3% (95% PI, 3.8%-8.8%) decrease from the expected number of cases. Overall, we estimated that the observed all-sites cancer incidence rate for the study period (March-December 2020) was 13.0% (95% PI, 11.2%-14.9%) lower than the expected rate of 375.4 cases per 100 000 (95% PI, 367.5-383.5). This equates to 134 395 (95% PI, 112 544-156 680) potentially missed cancer diagnoses in the US during the first 10 months of the COVID-19 pandemic.

**Table 2.** Potentially Missed All-Site and Screenable Cancer Cases, by Study Period During the COVID-19 Pandemic (March 1 Through December 31, 2020), USCS Public Use Database^8^

Cancer type and period	Observed rate^a^	Expected rate (95% PI)	Relative difference (95% PI)	Potential missed cases, No. (95% PI)
**All cancer sites**				
March-May	80.7	113.1 (108.2 to 118.2)	−28.6 (−31.7 to −25.4)^b^	88 830 (75 568 to 102 837)
June-December	245.8	262.4 (255.4 to 269.4)	−6.3 (−8.8 to −3.8)^b^	45 565 (26 479 to 65 032)
March-December	326.5	375.4 (367.5 to 383.5)	−13.0 (−14.9 to −11.2)^b^	134 395 (112 544 to 156 680)
**Screenable cancers^c^**				
March-May	28.6	41.3 (39.7 to 43.0)	−30.9 (−33.6 to −28.0)^b^	35 032 (30 564 to 39 710)
June-December	89.5	95.8 (93.5 to 98.2)	−6.6 (−8.8 to −4.3)^b^	17 298 (10 918 to 23 806)
March-December	118.1	137.1 (134.5 to 139.9)	−13.9 (−15.6 to −12.2)^b^	52 330 (45 034 to 59 767)

Abbreviations: PI, prediction interval; USCS, US Cancer Statistics.

^a^ Rates per 100 000 people in the population and age-adjusted to the 2000 US standard population.

^b^ Statistically significant disruption in observed vs expected incidence rates, based on 95% PI not including 0.

^c^ Screenable cancers were defined as female breast, lung and bronchus, colon and rectum, and cervix uteri.

**Figure 2.**
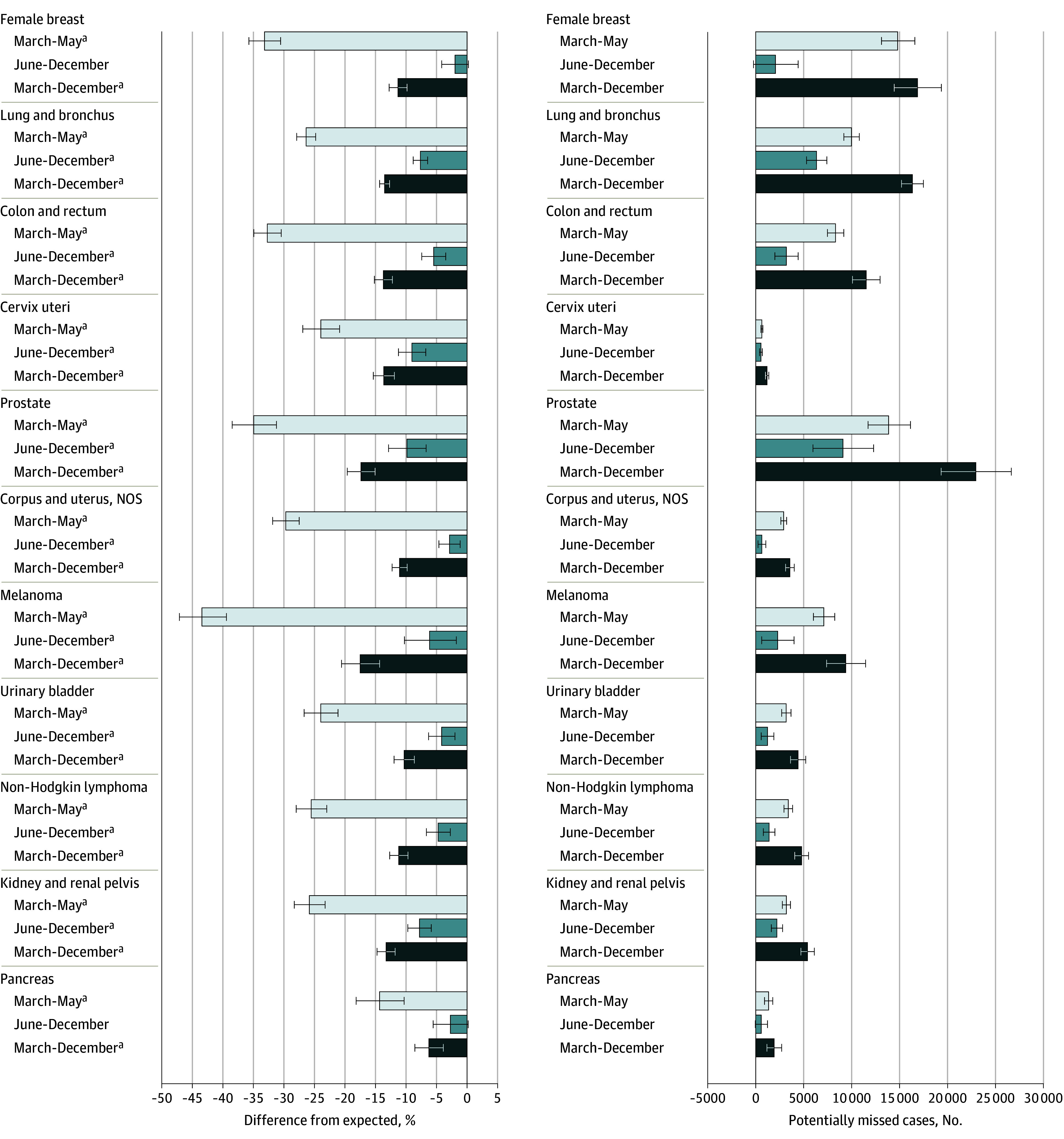
Percentage Difference Between Observed and Expected Incidence Rates and Potentially Missed Cancer Cases, by Cancer Site and Time Period, March 1 through December 31, 2020 Percentage difference in observed vs expected rates for each cancer site and time period (March-May 2020, June-December 2020, and March-December 2020) appear on the left. Estimated number of potential undiagnosed cases for each cancer site and period appear on the right. Error bars indicate the 95% PI (prediction interval). NOS indicates not otherwise specified. ^a^Indicates statistically significant disruption based on 95% PIs not containing 0.

We also fit models to 2018 to 2020 incidence rates for 11 cancer subsites and the combined rate across the 4 screenable cancers (Table 2, Figure 2; eTables 1-7 in Supplement 1). Every cancer site considered experienced statistically significant disruptions during March to May 2020. The greatest disruption during this period was found in melanoma diagnoses, which were 43.4% (95% PI, 39.4% to 47.1%) lower than expected. Statistically significant reductions continued from June to December 2020 for all sites except female breast (2.0% lower; 95% PI, 4.1% lower to 0.2% higher) and pancreatic (2.7% lower; 95% PI, 5.6% lower to 0.2% higher) cancers. Overall, observed cancer subsite incidence rates ranged from 6.2% (95% PI, 3.9% to 8.5%) lower than expected for pancreatic cancer to 17.5% (95% PI, 14.3% to 20.6%) lower than expected for melanoma in March to December 2020. We estimate that during March to December 2020, prostate cancer accounted for the largest number of potentially missed cases (22 950 cases; 95% PI, 19 337-26 646), followed by female breast cancer (16 870 cases; 95% PI, 14 438-19 360) and lung cancer (16 333 cases; 95% PI, 15 203-17 490).

### Population Cancer Rates by Stage at Diagnosis

**Figure 3.**
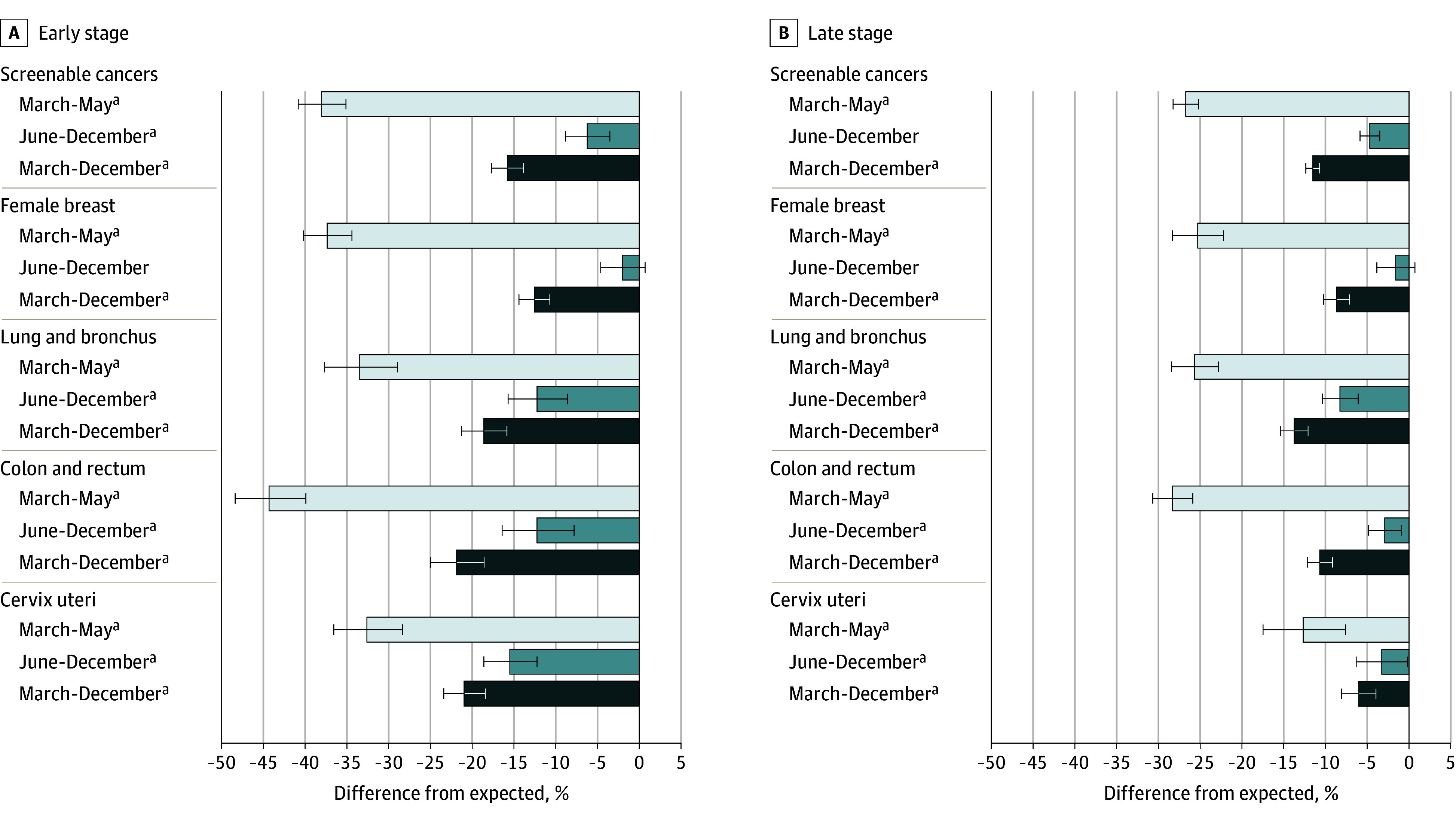
Percentage Difference Between Observed and Expected Incidence Rates for Screenable Cancers, by Site, Stage, and Time Period, March 1 Through December 31, 2020 Percentage difference in observed vs expected rates for each site and time period (March-May 2020, June-December 2020, and March-December 2020) according to early (left) or late (right) stage at cancer diagnosis. Error bars indicate 95% PI (prediction interval). ^a^Indicates statistically significant disruption based on 95% PI not containing 0.

During the study period (March-December 2020), observed rates of screenable cancers were 13.9% (95% PI, 12.2% to 15.6%) lower than expected. Early-stage diagnoses were lower than expected: 38.0% (95% PI, 35.1% to 40.8%) lower for March to May 2020; 6.3% (95% PI, 3.6% to 8.9%) lower for June to December 2020; and 15.8% (95% PI, 13.9% to 17.7%) lower for the full study period (March-December 2020) (Figure 3). Early-stage disruptions were comparable for lung, colorectal, and cervical cancers, and significantly less for female breast cancer. Given the apparent underdetection of early-stage cases, we assessed late-stage screenable cancers to determine whether there were any gains over the expected rate. Yet late-stage incidence rates were also lower than expected during the 3 study periods, respectively: 26.7% (95% PI, 25.2%-28.2%), 4.7% (95% PI, 3.5%-5.9%), and 11.5% (95% PI, 10.7%-12.3%) lower. Disruptions to late-stage lung cancer diagnoses were significantly higher than for female breast and cervical cancers, but comparable with disruptions in late-stage colorectal cancer incidence. The observation of rate recovery in female breast cancer diagnoses during June to December 2020 held true for both early-stage (2% lower; 95% PI, 4.7% lower to 0.6% higher) and late-stage (1.6% lower; 95% PI, 3.8% lower to 0.7% higher) disease.

All nonscreenable cancers had statistically significant early- and late-stage disruptions from March 1 through December 31, 2020. The most noticeable disruptions occurred in early-stage prostate cancer diagnoses, which were 20.8% (95% PI, 19.1%-22.5%) lower than expected, and early-stage melanoma diagnoses, which were 19.4% (95% PI, 16.2%-22.5%) lower than expected (eFigure 2 in Supplement 1).

### Population Subgroup Cancer Rates

From March 1 to May 31, 2020, all-sites cancer incidence rates had significantly worse disruptions among those living in states with more restrictive COVID-19 responses, and among individuals aged 65 years or older, but these differences did not persist when evaluated across the whole pandemic period of 2020 (eFigure 3 in Supplement 1). When evaluating the 11 cancer sites individually, 7 had significantly greater disruptions during the early pandemic in states with more restrictive responses, with significant disparity in disruptions during the total study period from March to December 2020 for lung, kidney, and pancreatic cancers (eFigure 4 in Supplement 1).

Other notable subgroup differences (eFigures 5 to 8 in Supplement 1) were found in lung cancer diagnoses, which had significantly greater disruptions among female than male patients during March to December 2020, and among older individuals, a subgroup that experienced significantly worse disruptions in screenable cancer rates. Rates of female breast cancer incidence appear to have returned to prepandemic trends during June to December 2020 for all population subgroups except White females and those living in states with less restrictive COVID-19 responses; among the other 3 screenable cancer sites, no applicable subgroup had rates return to expected levels during the last 7 months of 2020. Only kidney cancer showed a statistically significant disparity based on Black compared with White race, with greater disruptions among the Black population during March to May 2020 and for the whole study period. We found minimal differences based on urbanicity, with the only statistically significant disparity from March to December 2020 occurring for bladder cancer among metropolitan residents.

## Discussion

To our knowledge, this is the first study to offer a nationwide analysis using US cancer registry data on the cancer case deficit experienced during the COVID-19 pandemic in 2020. Our findings estimate that more than 134 000 cancer cases were undetected from March to December 2020—approximately, 1 missed case per 9 cases diagnosed—and that missed diagnoses differentially affected certain cancer sites, stages of diagnosis, and population subgroups. The findings from this analysis can inform the US health care system as decisions are made to recover the deficit through focused cancer screening and detection. These findings may also assist with planning for any future disruptions that would otherwise affect the timeliness of cancer diagnosis.

Much has been written about missed cancer screenings during 2020 and how they may affect incidence and mortality of screenable cancer sites in the coming years.^6,15,16,17^ Relying on data from the Behavioral Risk Factor Surveillance System (US Centers for Disease Control and Prevention) for 2018 and 2020, Fedewa et al^18^ found that the number of breast and cervical cancer screenings decreased slightly during the pandemic, while stool-based colon cancer testing increased and offset decreases in colonoscopy screening. Those findings gave hope because although underdetection was expected across all sites, colorectal cancer diagnoses may not have been significantly affected. However, using observed rates, we found substantial reductions in both early- and late-stage colorectal cancer incidence in March to December 2020, and significantly more than in early-stage breast and late-stage cervical cancers, respectively. Although our study did not span a time period sufficient for fully evaluating the repercussions of delayed screenings associated with the COVID-19 pandemic, this finding could indicate widespread delays in follow-up colonoscopy procedures after positive stool-based tests. Conversely, evidence of rate recovery for female breast cancer after the most restrictive pandemic response period suggests a resilience in mammography screening habits, likely borne from years of investment in public health messaging. It is important that we establish policies to help reengage with all patients regarding eligible screenings and necessary follow-ups, and work with patients to develop and maintain good cancer screening habits.

For cancers without high-evidence screenings, the literature assessing the association of the COVID-19 pandemic with diagnoses has relied largely on health system data.^19,20,21,22,23^ The well-documented reductions in health care utilization during the last 10 months of 2020 meant there were fewer opportunities for primary care physicians, dentists, and even rehabilitation therapy professionals to observe signs in a patient that may lead to a cancer diagnosis.^24,25^ An association between the 2 would be consistent with our findings of large differences between the expected rates of melanoma and prostate cancer incidence, as well as statistically significant differences associated with the extent of state-level COVID-19 restrictions and incidence of kidney and pancreatic cancers. Quantifying the association of pandemic restrictions with decreased office visits is as important as quantifying the contribution of missed screenings—it highlights the role of all medical professionals in detecting cancer. Our findings can help inform policies aimed at reducing missed appointments—eg, increased recruitment of nurses and clinical staff and targeted public health messaging—to assist patients with returning to routine visits across the health care landscape.

It is important that we continue to evaluate the trends identified in this study as US cancer incidence data for years after 2020 become available. Pandemic-associated disruptions will continue to affect rates of cancer incidence, and how long it will be until we fully recover is still unknown. Beyond incidence, it is important that we measure the pandemic’s contribution to future trends in cancer mortality and survival. With a near 10% reduction from expected rates in overall late-stage incidence from March to December 2020, there will undoubtedly—and unfortunately—be a subsequent rise in cancer mortality. How substantial a rise and for how long will provide a more complete picture of the consequences of COVID-19 disruptions on the burden of cancer in the US.

### Limitations

Although this study presents findings based on the most comprehensive and currently available data on trends in US cancer incidence, we acknowledge certain limitations. First, actual rates of cancer incidence may be higher than stated in the most recent periods due to delays in case reporting. Nevertheless, the magnitude of differences observed and the use of liberal prediction intervals for error bounds means the primary conclusions are unlikely to change meaningfully if data were revised. Second, not all cases had an available month of diagnosis, and those missing this information were excluded from the analysis; however, this affected no more than 0.5% of cases in any of the years considered, and there is no evidence of bias toward any particular month having greater amounts of incomplete data. Third, the number of potentially missed cases presented was based on a single value for the standard population for 2020 and thus did not adequately account for population changes within the year. Fourth, the dataset used had limited variables for items such as socioeconomic status, preventing more in-depth subgroup analyses. Fifth, this study classified the COVID-19 response of a patient’s state of residence based only on state-level stay-at-home-orders. There were several other factors independent of this specific intervention during March to December 2020 that may have affected individual behavior. Sixth, any analysis including age must consider the differential role of COVID-19 mortality on older individuals in 2020. Lastly, and with respect to statistical methods, we must note that postulated interruptions in the model were designed to capture the essence of observations from the raw data and aid in interpretation of results while not overfitting trends. Different interruptions could be postulated for specific trends that may have resulted in a better fit, but with a detriment to interpretation and broad applicability.

## Conclusions

The US experienced significant differences between observed and expected cancer incidence rates during the COVID-19 pandemic in 2020, with an associated and substantial deficit in diagnosed cases. The extent of differences varied based on cancer site, stage at diagnosis, and patient age and sex. These findings offer crucial information for current cancer prevention and control initiatives. Furthermore, these findings emphasize the need to consider how future disaster planning could affect cancer detection.

## Notes

Supplement 1. eMethods. Supplemental Description of Methods eTable 1. Study Population, Screenable Cancers and Female Breast Cancer Cases, January 2018-December 2020, USCS Public Use Database, 2001-2020 eTable 2. Study Population, Lung and Bronchus Cancer and Colon and Rectum Cancer Cases, January 2018-December 2020, USCS Public Use Database, 2001-2020 eTable 3. Study Population, Cervical Cancer and Prostate Cancer Cases, January 2018-December 2020, USCS Public Use Database, 2001-2020 eTable 4. Study Population, Corpus and Uterus, NOS Cancer and Melanoma Cases, January 2018-December 2020, USCS Public Use Database, 2001-2020 eTable 5. Study Population, Urinary Bladder Cancer and Non-Hodgkin Lymphoma Cases, January 2018-December 2020, USCS Public Use Database, 2001-2020 eTable 6. Study Population, Kidney and Renal Pelvis Cancer and Pancreas Cancer Cases, January 2018-December 2020, USCS Public Use Database, 2001-2020 eTable 7. Potentially Missed Cancer Cases, by Site and Period, March-December 2020, USCS Public Use Database, 2001-2020 eFigure 1. Study Population Sample Flow Diagram for All Sites Cancer Cases eFigure 2. Percentage Difference in Observed from Expected Incidence Rates for Non-Screenable Cancers, by Stage and Period, March-December 2020 eFigure 3. Percentage Difference in Observed from Expected All Sites Cancer Incidence Rates, by Population Subgroup and Period, March-December 2020 eFigure 4. Percentage Difference in Observed from Expected Incidence Rates, by COVID Response, Site, and Period, March-December 2020. eFigure 5. Percentage Difference in Observed from Expected Incidence Rates, by Urbanicity, Site, and Period, March-December 2020 eFigure 6. Percentage Difference in Observed from Expected Incidence Rates, by Age, Site, and Period, March-December 2020 eFigure 7. Percentage Difference in Observed from Expected Incidence Rates, by Race, Site, and Period, March-December 2020 eFigure 8. Percentage Difference in Observed from Expected Incidence Rates, by Sex, Site, and Period, March-December 2020

Supplement 2. Data Sharing Statement
