# Supplementary material for: Undiagnosed Cancer Cases in the US During the First 10 Months of the COVID-19 Pandemic
Source: JAMA Oncol. 2024 Feb 22;10(4):500–7. doi: 10.1001/jamaoncol.2023.6969 (PMC10884945; doi:10.1001/jamaoncol.2023.6969)
Supplement: Supplement 1. — eMethods. Supplemental Description of Methods eTable 1. Study Population, Screenable Cancers and Female Breast Cancer Cases, January 2018-December 2020, USCS Public Use Database, 2001-2020 eTable 2. Study Population, Lung and Bronchus Cancer and Colon and Rectum Cancer Cases, January 2018-December 2020, USCS Public Use Database, 2001-2020 eTable 3. Study Population, Cervical Cancer and Prostate Cancer Cases, January 2018-December 2020, USCS Public Use Database, 2001-2020 eTable 4. Study Population, Corpus and Uterus, NOS Cancer and Melanoma Cases, January 2018-December 2020, USCS Public Use Database, 2001-2020 eTable 5. Study Population, Urinary Bladder Cancer and Non-Hodgkin Lymphoma Cases, January 2018-December 2020, USCS Public Use Database, 2001-2020 eTable 6. Study Population, Kidney and Renal Pelvis Cancer and Pancreas Cancer Cases, January 2018-December 2020, USCS Public Use Database, 2001-2020 eTable 7. Potentially Missed Cancer Cases, by Site and Period, March-December 2020, USCS Public Use Database, 2001-2020 eFigure 1. Study Population Sample Flow Diagram for All Sites Cancer Cases eFigure 2. Percentage Difference in Observed from Expected Incidence Rates for Non-Screenable Cancers, by Stage and Period, March-December 2020 eFigure 3. Percentage Difference in Observed from Expected All Sites Cancer Incidence Rates, by Population Subgroup and Period, March-December 2020 eFigure 4. Percentage Difference in Observed from Expected Incidence Rates, by COVID Response, Site, and Period, March-December 2020. eFigure 5. Percentage Difference in Observed from Expected Incidence Rates, by Urbanicity, Site, and Period, March-December 2020 eFigure 6. Percentage Difference in Observed from Expected Incidence Rates, by Age, Site, and Period, March-December 2020 eFigure 7. Percentage Difference in Observed from Expected Incidence Rates, by Race, Site, and Period, March-December 2020 eFigure 8. Percentage Difference in Observed from Expected Incidence Rates, by Sex, Site, [file jamaoncol-e236969-s001.pdf]

Supplementary Online Content

Burus T, Lei F, Huang B, et al. Undiagnosed Cancer Cases in the US During the First 10 Months of the COVID-19 Pandemic. *JAMA Oncol*. Published online February 22, 2024. doi:10.1001/jamaoncol.2023.6969

eMethods. Supplemental Description of Methods

eTable 1. Study Population, Screenable Cancers and Female Breast Cancer Cases, January 2018-December 2020, USCS Public Use Database, 2001-2020

eTable 2. Study Population, Lung and Bronchus Cancer and Colon and Rectum Cancer Cases, January 2018-December 2020, USCS Public Use Database, 2001-2020

eTable 3. Study Population, Cervical Cancer and Prostate Cancer Cases, January 2018-December 2020, USCS Public Use Database, 2001-2020

eTable 4. Study Population, Corpus and Uterus, NOS Cancer and Melanoma Cases, January 2018-December 2020, USCS Public Use Database, 2001-2020

eTable 5. Study Population, Urinary Bladder Cancer and Non-Hodgkin Lymphoma Cases, January 2018-December 2020, USCS Public Use Database, 2001-2020

eTable 6. Study Population, Kidney and Renal Pelvis Cancer and Pancreas Cancer Cases, January 2018-December 2020, USCS Public Use Database, 2001-2020

eTable 7. Potentially Missed Cancer Cases, by Site and Period, March-December 2020, USCS Public Use Database, 2001-2020

eFigure 1. Study Population Sample Flow Diagram for All Sites Cancer Cases

eFigure 2. Percentage Difference in Observed from Expected Incidence Rates for Non-Screenable Cancers, by Stage and Period, March-December 2020

eFigure 3. Percentage Difference in Observed from Expected All Sites Cancer Incidence Rates, by Population Subgroup and Period, March-December 2020

eFigure 4. Percentage Difference in Observed from Expected Incidence Rates, by COVID Response, Site, and Period, March-December 2020.

eFigure 5. Percentage Difference in Observed from Expected Incidence Rates, by Urbanicity, Site, and Period, March-December 2020

eFigure 6. Percentage Difference in Observed from Expected Incidence Rates, by Age, Site, and Period, March-December 2020

eFigure 7. Percentage Difference in Observed from Expected Incidence Rates, by Race, Site, and Period, March-December 2020

eFigure 8. Percentage Difference in Observed from Expected Incidence Rates, by Sex, Site, and Period, March-December 2020

This supplementary material has been provided by the authors to give readers additional information about their work

© 2024 Burus et al. JAMA Oncology.

eMethods. Supplemental Description of Methods

A. Seasonal ARIMA models, exogenous regressors, model fitting and forecasting.

In this study we forecasted cancer rates for March-December 2020 by first fitting (seasonal) autoregressive integrated moving average (ARIMA) models with exogenous regressors to observed monthly cancer rate trends from January 2018-December 2020. Basic ARIMA models are a linear combination of autoregressive models (lagged variables) and moving average models (lagged errors) with possible differencing of adjacent terms to induce stationarity. An ARIMA model is specified by the order of the autoregressive component (p), the order of the differencing used (d), and the order of the moving average component (q). Additional seasonal ARIMA parameters P, D, and Q can be added to handle seasonal trends for seasons of period length m. Any exogenous regressors are included in the final model as additive regression terms external to the ARIMA model defined.

For our models we postulated two exogenous regressors based on contextual knowledge of the progression of the COVID-19 pandemic in the United States during the year 2020 and a cursory inspection of collected trends. The first exogenous regressor was a pulse effect (a sudden, short-term change in trends) over the period of March-May 2020. This regressor was intended to capture the average change in rates from previous trends during the period of broad stay-at-home orders and was encoded as a vector taking the value 1 for March, April and May 2020 and 0 elsewhere. With this encoding, the exponential of the coefficient of the pulse effect regressor in the best fit model can be interpreted as the rate ratio between the mean observed incidence rate in March-May 2020 and the mean expected incidence rate in March-May 2020.

The second regressor was a step change effect (a sudden and sustained change in trends) over the period of June-December 2020. This regressor was intended to capture the average change in rates from pre-pandemic trends following the conclusion of the period of broad stay-at-home orders. The step change effect was encoded as a vector taking a value of 1 for every month from June-December 2020 and 0 elsewhere. Similar to the pulse effect regressor, encoding the step change effect regressor in this fashion allows us to interpret the exponential of the coefficient of it as the rate ratio between the mean observed incidence rate in June-December 2020 and the mean expected incidence rate in June-December 2020. It was unclear if this effect would prove significant for all or any of the incidence rate time series considered.

A ramp effect regressor (a linear increase or decrease in trends over time) was initially considered in place of a step change effect for the period of June-December 2020, however a basic visual inspection of trends collected contraindicated this.

The graph below illustrates a theoretical time series in which both a significant pulse effect and step change effect are observed:

© 2024 Burus et al. JAMA Oncology.

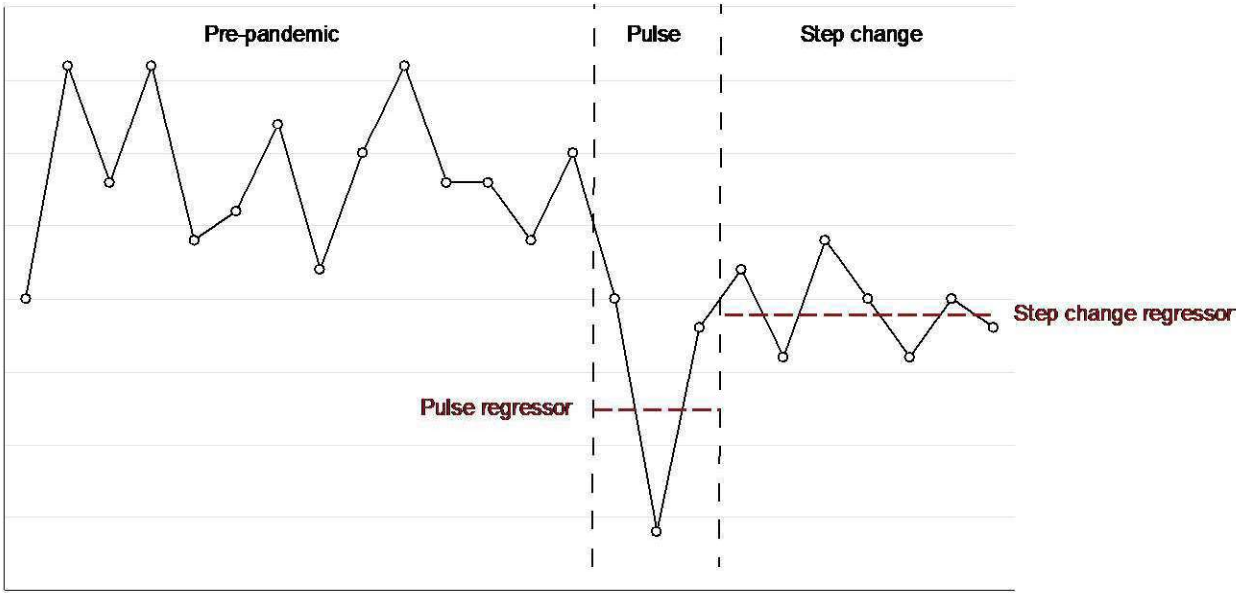

To select the best fit model for a given time series of monthly cancer incidence rates, we first applied a log transformation to rates to stabilize their variance. We then iteratively fit models with both the pulse and step change exogenous regressors using the auto.arima() function from the forecast package in R, allowing for maximum *d* and *D* parameters of 1.[1] The best fit model was selected based on minimum Akaike Information Criteria. A summary for the best fit model was produced and the presence of residual autocorrelation was checked using the Ljung-Box test. If the coefficient for the step change regressors failed to be significant, or if the Ljung-Box test indicated significant residual autocorrelation, then the model was re-fit without the step change regressor. This process was repeated to assess the significance of a pulse impact, which held for all models tested.

To forecast counterfactual rates assuming the COVID-19 pandemic had not occurred, we used the parameters of the best fit model for the full time series and applied them to fit an ARIMA model on the rates from January 2018-February 2020. Expected rates were then calculated for the periods of March-May 2020, June-December 2020, and March-December 2020 using simulations. We performed 10,000 forecasts of monthly rates for June-December 2020, taking the mean of the sum of the simulated rates (or an appropriate subset for the reduced periods) as a point estimate and the 2.5% and 97.5% percentiles of the sum of simulated rates (or an appropriate subset for the reduced periods) as a 95% prediction interval.[2] A random seed was set before running simulations to ensure reproducibility.

B. Determining severity of COVID response by state stay-at-home orders.

The severity of a state's COVID response was determined by analyzing the dataset 'OxCGRT_USA_differentiated_withnotes_2020.csv' produced by the Oxford Covid-19 Government Response Tracker (OxCGRT).[3,4] This dataset has been used to track national and sub-national responses to the COVID-19 pandemic since January 2020. In particular, we considered U.S. state level information about statewide stay-at-home requirements (variable 'C6E_Stay at home requirements', henceforth C6E). Dates for the beginning and end of a state's official statewide stay-at-home orders (if applicable) were determined by looking at dates between March 1, 2020 and May 31, 2020 having C6E value of 2 ("require not leaving house with exceptions for daily exercise, grocery shopping, and 'essential' trips") and a C6E_Flag value of 1 ("general"). It was found that the median start date of statewide stay-at-home orders across the U.S. was March 27, 2020 and the median end date was May 8, 2020—a period of 42 days. This length of time served as our cutoff for classifying a state's COVID response as being more (stay-at-homes orders for > 42 days) or less (stay-at-home orders ≤ 42 days) restrictive.

© 2024 Burus et al. JAMA Oncology.

Upon constructing the initial dataset, OxCGRT table notes were reviewed (specifically, variable 'C6_Notes') to determine if adjustments needed to be made to start or end dates for certain states. This process led us to revise the stay-at-home order dates for five states (Massachusetts, Nevada, New Mexico, North Carolina, and Vermont). The final values used for classifying a state's COVID response are listed in the table below:

State	Stay-at-home start	Stay-at-home end	Stay-at-home length
Alabama	4/4/2020	4/29/2020	25
Alaska	3/28/2020	4/23/2020	26
Arizona	3/31/2020	5/15/2020	45
Arkansas	n/a	n/a	0
California	3/19/2020	5/7/2020	49
Colorado	3/26/2020	4/26/2020	31
Connecticut	n/a	n/a	0
Delaware	3/24/2020	6/1/2020	69
Florida	4/3/2020	5/3/2020	30
Georgia	4/3/2020	4/23/2020	20
Hawaii	3/25/2020	6/1/2020	68
Idaho	3/25/2020	4/30/2020	36
Illinois	3/21/2020	6/1/2020	72
Indiana	3/25/2020	4/11/2020	17
Iowa	n/a	n/a	0
Kansas	3/30/2020	5/3/2020	34
Kentucky	3/26/2020	5/21/2020	56
Louisiana	3/23/2020	5/14/2020	52
Maine	4/2/2020	5/30/2020	58
Maryland	3/30/2020	5/13/2020	44
Massachusetts	3/23/2020	5/18/2020	56
Michigan	3/24/2020	5/17/2020	54
Minnesota	3/27/2020	5/17/2020	51
Mississippi	4/3/2020	5/31/2020	58
Missouri	4/6/2020	5/3/2020	27
Montana	3/28/2020	4/24/2020	27
Nebraska	n/a	n/a	0
Nevada	4/1/2020	5/15/2020	44
New Hampshire	3/27/2020	6/1/2020	66
New Jersey	3/21/2020	6/1/2020	72
New Mexico	3/23/2020	5/15/2020	53
New York	3/22/2020	5/14/2020	53
North Carolina	3/30/2020	5/22/2020	53
North Dakota	n/a	n/a	0
Ohio	3/23/2020	5/19/2020	57
Oklahoma	3/24/2020	4/23/2020	30

© 2024 Burus et al. JAMA Oncology.

Oregon	3/27/2020	5/14/2020	48
Pennsylvania	4/1/2020	5/8/2020	36
Rhode Island	3/28/2020	5/7/2020	40
South Carolina	4/7/2020	5/2/2020	25
South Dakota	n/a	n/a	0
Tennessee	3/30/2020	4/27/2020	28
Texas	4/2/2020	4/30/2020	28
Utah	n/a	n/a	0
Vermont	3/24/2020	5/15/2020	52
Virginia	3/30/2020	5/14/2020	45
Washington	3/23/2020	5/31/2020	69
Washington DC	4/1/2020	5/28/2020	57
West Virginia	3/23/2020	5/3/2020	41
Wisconsin	3/25/2020	5/12/2020	48
Wyoming	n/a	n/a	0

© 2024 Burus et al. JAMA Oncology.

eTable 1. Study Population, Screenable Cancers and Female Breast Cancer Cases, January 2018-December 2020, USCS Public Use Database, 2001-2020

	Screenable Cancers		Female Breast	
	Pre, N (%)	Pan, N (%)	Pre, N (%)	Pan, N (%)
Total	1376281	466080	562861	194092
Included [b]	1373124 (99.8)	465002 (99.8)	561754 (99.8)	193673 (99.8)
Missing month [c]	3157 (0.2)	1078 (0.2)	1107 (0.2)	419 (0.2)
Sex				
Male	404683 (29.5)	135471 (29.1)	-	-
Female	968441 (70.5)	329531 (70.9)	561754 (100)	193673 (100)
Age				
Under 65	585100 (42.6)	198721 (42.7)	291389 (51.9)	100534 (51.9)
65 or older	788024 (57.4)	266281 (57.3)	270365 (48.1)	93139 (48.1)
Race				
Black	161499 (11.8)	54711 (11.8)	67041 (11.9)	23594 (12.2)
White	1130832 (82.4)	380879 (81.9)	457482 (81.4)	156246 (80.7)
Other race [d]	80793 (5.9)	29412 (6.3)	37231 (6.6)	13833 (7.1)
Urbanicity				
Metropolitan	1112427 (81)	376426 (81)	468612 (83.4)	161455 (83.4)
Non-metropolitan	225201 (16.4)	76122 (16.4)	77970 (13.9)	26816 (13.8)
Unknown	35496 (2.6)	12454 (2.7)	15172 (2.7)	5402 (2.8)
Stage				
Early	615804 (44.8)	203667 (43.8)	372505 (66.3)	126920 (65.5)
Late	675287 (49.2)	234723 (50.5)	172055 (30.6)	60887 (31.4)
Unknown	82033 (6)	26612 (5.7)	17194 (3.1)	5866 (3)
COVID Response				
More restrictive	796556 (58)	267849 (57.6)	332906 (59.3)	113622 (58.7)
Less restrictive	576568 (42)	197153 (42.4)	228848 (40.7)	80051 (41.3)

[a] Pre-pandemic study period features cases used to build ARIMA models
[b] Denominator used for calculating subgroup percentages
[c] Cases missing month of diagnosis in 2020 were assigned to the pandemic study period
[d] Other race includes American Indian/Alaska Native, Asian or Pacific Islander, and Unknown
Abbreviations: Pre = Pre-pandemic study period (January 2018 – February 2020);
Pan = Pandemic study period (March – December 2020)

© 2024 Burus et al. JAMA Oncology.

eTable 2. Study Population, Lung and Bronchus Cancer and Colon and Rectum Cancer Cases, January 2018-December 2020, USCS Public Use Database, 2001-2020

	Lung and Bronchus		Colon and Rectum	
	Pre, N (%)	Pan, N (%)	Pre, N (%)	Pan, N (%)
Total	478698	160097	306894	102605
Included [b]	477496 (99.7)	159706 (99.8)	306176 (99.8)	102374 (99.8)
Missing month [c]	1202 (0.3)	391 (0.2)	718 (0.2)	231 (0.2)
Sex				
Male	242227 (50.7)	81413 (51)	162456 (53.1)	54058 (52.8)
Female	235269 (49.3)	78293 (49)	143720 (46.9)	48316 (47.2)
Age				
Under 65	138329 (29)	45671 (28.6)	133490 (43.6)	45306 (44.3)
65 or older	339167 (71)	114035 (71.4)	172686 (56.4)	57068 (55.7)
Race				
Black	51844 (10.9)	16997 (10.6)	38310 (12.5)	12750 (12.5)
White	404775 (84.8)	135293 (84.7)	247666 (80.9)	82403 (80.5)
Other race [d]	20877 (4.4)	7416 (4.6)	20200 (6.6)	7221 (7.1)
Urbanicity				
Metropolitan	375483 (78.6)	125224 (78.4)	245502 (80.2)	82136 (80.2)
Non-metropolitan	89931 (18.8)	30239 (18.9)	53094 (17.3)	17637 (17.2)
Unknown	12082 (2.5)	4243 (2.7)	7580 (2.5)	2601 (2.5)
Stage				
Early	131091 (27.5)	42617 (26.7)	100209 (32.7)	30494 (29.8)
Late	307891 (64.5)	105067 (65.8)	181648 (59.3)	63821 (62.3)
Unknown	38514 (8.1)	12022 (7.5)	24319 (7.9)	8059 (7.9)
COVID Response				
More restrictive	272803 (57.1)	90964 (57)	175674 (57.4)	58295 (56.9)
Less restrictive	204693 (42.9)	68742 (43)	130502 (42.6)	44079 (43.1)

[a] Pre-pandemic study period features cases used to build ARIMA models
[b] Denominator used for calculating subgroup percentages
[c] Cases missing month of diagnosis in 2020 were assigned to the pandemic study period
[d] Other race includes American Indian/Alaska Native, Asian or Pacific Islander, and Unknown
Abbreviations: Pre = Pre-pandemic study period (January 2018 – February 2020);
Pan = Pandemic study period (March – December 2020)

© 2024 Burus et al. JAMA Oncology.

eTable 3. Study Population, Cervical Cancer and Prostate Cancer Cases, January 2018-December 2020, USCS Public Use Database, 2001-2020

	Cervix Uteri		Prostate	
	Pre, N (%)	Pan, N (%)	Pre, N (%)	Pan, N (%)
Total	27828	9286	491660	160521
Included [b]	27698 (99.5)	9249 (99.6)	489165 (99.5)	159612 (99.4)
Missing month [c]	130 (0.5)	37 (0.4)	2495 (0.5)	909 (0.6)
Sex				
Male	-	-	489165 (100)	159612 (100)
Female	27698 (100)	9249 (100)	-	-
Age				
Under 65	21892 (79)	7210 (78)	181811 (37.2)	56345 (35.3)
65 or older	5806 (21)	2039 (22)	307354 (62.8)	103267 (64.7)
Race				
Black	4304 (15.5)	1370 (14.8)	78666 (16.1)	25313 (15.9)
White	20909 (75.5)	6937 (75)	379172 (77.5)	122258 (76.6)
Other race [d]	2485 (9)	942 (10.2)	31327 (6.4)	12041 (7.5)
Urbanicity				
Metropolitan	22830 (82.4)	7611 (82.3)	400498 (81.9)	130994 (82.1)
Non-metropolitan	4206 (15.2)	1430 (15.5)	76546 (15.6)	24471 (15.3)
Unknown	662 (2.4)	208 (2.2)	12121 (2.5)	4147 (2.6)
Stage				
Early	11999 (43.3)	3636 (39.3)	342480 (70)	107084 (67.1)
Late	13693 (49.4)	4948 (53.5)	102976 (21.1)	35988 (22.5)
Unknown	2006 (7.2)	665 (7.2)	43709 (8.9)	16540 (10.4)
COVID Response				
More restrictive	15173 (54.8)	4968 (53.7)	289165 (59.1)	94763 (59.4)
Less restrictive	12525 (45.2)	4281 (46.3)	200000 (40.9)	64849 (40.6)

[a] Pre-pandemic study period features cases used to build ARIMA models
[b] Denominator used for calculating subgroup percentages
[c] Cases missing month of diagnosis in 2020 were assigned to the pandemic study period
[d] Other race includes American Indian/Alaska Native, Asian or Pacific Islander, and Unknown
Abbreviations: Pre = Pre-pandemic study period (January 2018 – February 2020);
Pan = Pandemic study period (March – December 2020)

© 2024 Burus et al. JAMA Oncology.

eTable 4. Study Population, Corpus and Uterus, NOS Cancer and Melanoma Cases, January 2018-December 2020, USCS Public Use Database, 2001-2020

	Corpus and Uterus, NOS		Melanoma	
	Pre, N (%)	Pan, N (%)	Pre, N (%)	Pan, N (%)
Total	126875	44717	188718	62410
Included [b]	126625 (99.8)	44632 (99.8)	188150 (99.7)	62200 (99.7)
Missing month [c]	250 (0.2)	85 (0.2)	568 (0.3)	210 (0.3)
Sex				
Male	-	-	111393 (59.2)	36317 (58.4)
Female	126625 (100)	44632 (100)	76757 (40.8)	25883 (41.6)
Age				
Under 65	65966 (52.1)	22744 (51)	85143 (45.3)	27694 (44.5)
65 or older	60659 (47.9)	21888 (49)	103007 (54.7)	34506 (55.5)
Race				
Black	16152 (12.8)	5915 (13.3)	826 (0.4)	286 (0.5)
White	101939 (80.5)	35452 (79.4)	176764 (93.9)	57379 (92.2)
Other race [d]	8534 (6.7)	3265 (7.3)	10560 (5.6)	4535 (7.3)
Urbanicity				
Metropolitan	104941 (82.9)	36924 (82.7)	154711 (82.2)	50745 (81.6)
Non-metropolitan	18391 (14.5)	6470 (14.5)	28489 (15.1)	9793 (15.7)
Unknown	3293 (2.6)	1238 (2.8)	4950 (2.6)	1662 (2.7)
Stage				
Early	87327 (69)	30182 (67.6)	144650 (76.9)	46610 (74.9)
Late	32869 (26)	12350 (27.7)	25858 (13.7)	9565 (15.4)
Unknown	6429 (5.1)	2100 (4.7)	17642 (9.4)	6025 (9.7)
COVID Response				
More restrictive	76389 (60.3)	26946 (60.4)	112053 (59.6)	36949 (59.4)
Less restrictive	50236 (39.7)	17686 (39.6)	76097 (40.4)	25251 (40.6)

[a] Pre-pandemic study period features cases used to build ARIMA models
[b] Denominator used for calculating subgroup percentages
[c] Cases missing month of diagnosis in 2020 were assigned to the pandemic study period
[d] Other race includes American Indian/Alaska Native, Asian or Pacific Islander, and Unknown
Abbreviations: Pre = Pre-pandemic study period (January 2018 – February 2020);
Pan = Pandemic study period (March – December 2020); NOS = Not otherwise specified

© 2024 Burus et al. JAMA Oncology.

eTable 5. Study Population, Urinary Bladder Cancer and Non-Hodgkin Lymphoma Cases, January 2018-December 2020, USCS Public Use Database, 2001-2020

	Urinary Bladder		Non-Hodgkin Lymphoma	
	Pre, N (%)	Pan, N (%)	Pre, N (%)	Pan, N (%)
Total	162732	57484	156180	54576
Included [b]	162299 (99.7)	57354 (99.8)	155734 (99.7)	54425 (99.7)
Missing month [c]	433 (0.3)	130 (0.2)	446 (0.3)	151 (0.3)
Sex				
Male	123666 (76.2)	43932 (76.6)	86117 (55.3)	30188 (55.5)
Female	38633 (23.8)	13422 (23.4)	69617 (44.7)	24237 (44.5)
Age				
Under 65	38342 (23.6)	13219 (23)	61342 (39.4)	20791 (38.2)
65 or older	123957 (76.4)	44135 (77)	94392 (60.6)	33634 (61.8)
Race				
Black	9927 (6.1)	3509 (6.1)	12882 (8.3)	4349 (8)
White	145826 (89.9)	51173 (89.2)	133231 (85.6)	46338 (85.1)
Other race [d]	6546 (4)	2672 (4.7)	9621 (6.2)	3738 (6.9)
Urbanicity				
Metropolitan	130936 (80.7)	46139 (80.4)	128839 (82.7)	44904 (82.5)
Non-metropolitan	27473 (16.9)	9770 (17)	23221 (14.9)	8143 (15)
Unknown	3890 (2.4)	1445 (2.5)	3674 (2.4)	1378 (2.5)
Stage				
Early	57096 (35.2)	20668 (36)	37810 (24.3)	12917 (23.7)
Late	20045 (12.4)	7668 (13.4)	99338 (63.8)	35754 (65.7)
Unknown	85158 (52.5)	29018 (50.6)	18586 (11.9)	5754 (10.6)
COVID Response				
More restrictive	95702 (59)	33773 (58.9)	91145 (58.5)	31737 (58.3)
Less restrictive	66597 (41)	23581 (41.1)	64589 (41.5)	22688 (41.7)

[a] Pre-pandemic study period features cases used to build ARIMA models
[b] Denominator used for calculating subgroup percentages
[c] Cases missing month of diagnosis in 2020 were assigned to the pandemic study period
[d] Other race includes American Indian/Alaska Native, Asian or Pacific Islander, and Unknown
Abbreviations: Pre = Pre-pandemic study period (January 2018 – February 2020);
Pan = Pandemic study period (March – December 2020)

© 2024 Burus et al. JAMA Oncology.

eTable 6. Study Population, Kidney and Renal Pelvis Cancer and Pancreas Cancer Cases, January 2018-December 2020, USCS Public Use Database, 2001-2020

	Kidney and Renal Pelvis		Pancreas	
	Pre, N (%)	Pan, N (%)	Pre, N (%)	Pan, N (%)
Total	147030	50796	116525	43442
Included [b]	146690 (99.8)	50679 (99.8)	116141 (99.7)	43328 (99.7)
Missing month [c]	340 (0.2)	117 (0.2)	384 (0.3)	114 (0.3)
Sex				
Male	93433 (63.7)	32792 (64.7)	60211 (51.8)	22639 (52.3)
Female	53257 (36.3)	17887 (35.3)	55930 (48.2)	20689 (47.7)
Age				
Under 65	70548 (48.1)	24082 (47.5)	36032 (31)	13117 (30.3)
65 or older	76142 (51.9)	26597 (52.5)	80109 (69)	30211 (69.7)
Race				
Black	18073 (12.3)	6108 (12.1)	14995 (12.9)	5497 (12.7)
White	121155 (82.6)	41746 (82.4)	95182 (82)	35422 (81.8)
Other race [d]	7462 (5.1)	2825 (5.6)	5964 (5.1)	2409 (5.6)
Urbanicity				
Metropolitan	118346 (80.7)	40898 (80.7)	94882 (81.7)	35527 (82)
Non-metropolitan	24756 (16.9)	8505 (16.8)	18267 (15.7)	6665 (15.4)
Unknown	3588 (2.4)	1276 (2.5)	2992 (2.6)	1136 (2.6)
Stage				
Early	98480 (67.1)	33182 (65.5)	19332 (16.6)	7239 (16.7)
Late	41263 (28.1)	15322 (30.2)	84374 (72.6)	31920 (73.7)
Unknown	6947 (4.7)	2175 (4.3)	12435 (10.7)	4169 (9.6)
COVID Response				
More restrictive	83462 (56.9)	28371 (56)	68738 (59.2)	25231 (58.2)
Less restrictive	63228 (43.1)	22308 (44)	47403 (40.8)	18097 (41.8)

[a] Pre-pandemic study period features cases used to build ARIMA models
[b] Denominator used for calculating subgroup percentages
[c] Cases missing month of diagnosis in 2020 were assigned to the pandemic study period
[d] Other race includes American Indian/Alaska Native, Asian or Pacific Islander, and Unknown
Abbreviations: Pre = Pre-pandemic study period (January 2018 – February 2020);
Pan = Pandemic study period (March – December 2020)

© 2024 Burus et al. JAMA Oncology.

eTable 7. Potentially Missed Cancer Cases, by Site and Period, March-December 2020, USCS Public Use Database, 2001-2020[5]

	Period	Observed Rate[a]	Expected Rate (95% PI)	Relative Difference (95% PI)	Potential Missed Cases (95% PI)
Female Breast	Mar-May	21.7	32.5 (31.3, 33.8)	-33.2% (-35.7%, -30.5%)*	14805 (13115, 16591)
	June-Dec	74.7	76.2 (74.5, 77.9)	-2.0% (-4.1%, 0.2%)	2065 (-227, 4432)
	Mar-Dec	96.4	108.7 (106.9, 110.5)	-11.3% (-12.8%, -9.8%)*	16870 (14438, 19360)
Lung and Bronchus	Mar-May	10.2	13.8 (13.5, 14.1)	-26.4% (-27.9%, -24.8%)*	9986 (9196, 10803)
	June-Dec	28.0	30.3 (29.9, 30.7)	-7.6% (-8.8%, -6.5%)*	6346 (5300, 7423)
	Mar-Dec	38.1	44.1 (43.7, 44.5)	-13.5% (-14.3%, -12.7%)*	16333 (15203, 17490)
Colon and Rectum	Mar-May	6.2	9.3 (9.0, 9.6)	-32.7% (-34.9%, -30.4%)*	8321 (7485, 9187)
	June-Dec	20.2	21.3 (20.9, 21.8)	-5.5% (-7.4%, -3.5%)*	3200 (2000, 4428)
	Mar-Dec	26.4	30.6 (30.1, 31.1)	-13.7% (-15.2%, -12.2%)*	11521 (10095, 12968)
Cervix Uteri	Mar-May	1.5	2.0 (1.9, 2.0)	-23.9% (-26.9%, -20.9%)*	643 (539, 752)
	June-Dec	4.0	4.4 (4.3, 4.5)	-9.0% (-11.2%, -6.8%)*	542 (396, 691)
	Mar-Dec	5.5	6.3 (6.2, 6.5)	-13.6% (-15.3%, -11.9%)*	1186 (1014, 1362)
Prostate	Mar-May	18.8	28.9 (27.3, 30.5)	-34.9% (-38.5%, -31.2%)*	13856 (11712, 16138)
	June-Dec	60.7	67.3 (65, 69.6)	-9.8% (-12.9%, -6.7%)*	9094 (5975, 12293)
	Mar-Dec	79.5	96.2 (93.5, 98.9)	-17.4% (-19.6%, -15.1%)*	22950 (19337, 26646)
Corpus and Uterus, NOS	Mar-May	5.0	7.1 (6.9, 7.4)	-29.7% (-31.9%, -27.5%)*	2916 (2617, 3226)
	June-Dec	15.8	16.3 (16.0, 16.6)	-2.9% (-4.6%, -1.1%)*	643 (248, 1051)
	Mar-Dec	20.9	23.5 (23.1, 23.8)	-11.0% (-12.3%, -9.8%)*	3559 (3115, 4013)
Melanoma	Mar-May	3.4	6 (5.6, 6.4)	-43.4% (-47.1%, -39.4%)*	7098 (6016, 8241)
	June-Dec	12.8	13.6 (13.0, 14.2)	-6.1% (-10.3%, -1.8%)*	2292 (626, 4003)
	Mar-Dec	16.1	19.5 (18.8, 20.3)	-17.5% (-20.6%, -14.3%)*	9390 (7393, 11459)
Urinary Bladder	Mar-May	3.7	4.8 (4.7, 5.0)	-24.0% (-26.7%, -21.1%)*	3185 (2709, 3679)
	June-Dec	10.3	10.8 (10.5, 11.0)	-4.2% (-6.3%, -2.0%)*	1230 (571, 1901)
	Mar-Dec	14.0	15.6 (15.3, 15.9)	-10.3% (-12.0%, -8.6%)*	4415 (3630, 5214)
Non-Hodgkin Lymphoma	Mar-May	3.6	4.8 (4.7, 5.0)	-25.5% (-28.0%, -23.0%)*	3394 (2953, 3847)
	June-Dec	10.2	10.8 (10.5, 11)	-4.7% (-6.7%, -2.7%)*	1393 (793, 2004)
	Mar-Dec	13.8	15.6 (15.3, 15.9)	-11.2% (-12.7%, -9.7%)*	4787 (4072, 5516)
Kidney and Renal Pelvis	Mar-May	3.3	4.5 (4.4, 4.7)	-25.8% (-28.3%, -23.3%)*	3196 (2781, 3623)
	June-Dec	9.5	10.3 (10.1, 10.6)	-7.8% (-9.7%, -5.9%)*	2213 (1627, 2810)
	Mar-Dec	12.9	14.8 (14.6, 15.1)	-13.3% (-14.7%, -11.8%)*	5408 (4716, 6115)
Pancreas	Mar-May	2.9	3.4 (3.3, 3.6)	-14.3% (-18.2%, -10.3%)*	1346 (925, 1787)
	June-Dec	7.6	7.8 (7.6, 8.1)	-2.7% (-5.6%, 0.2%)	585 (41, 1232)
	Mar-Dec	10.6	11.3 (11.0, 11.5)	-6.2% (-8.5%, -3.9%)*	1931 (1173, 2706)

* Significant disruption in observed versus expected incidence rates, based on 95% prediction interval not including 0

[a] Rates given per 100,000 people in the population and age-adjusted to the 2000 U.S. Standard Population

Abbreviations: PI = Prediction Interval; Mar = March; Dec = December

© 2024 Burus et al. JAMA Oncology.

eFigure 1. Study population sample flow diagram for All Sites Cancer Cases.
Similar process repeated for 11 site-specific cancers analyzed.

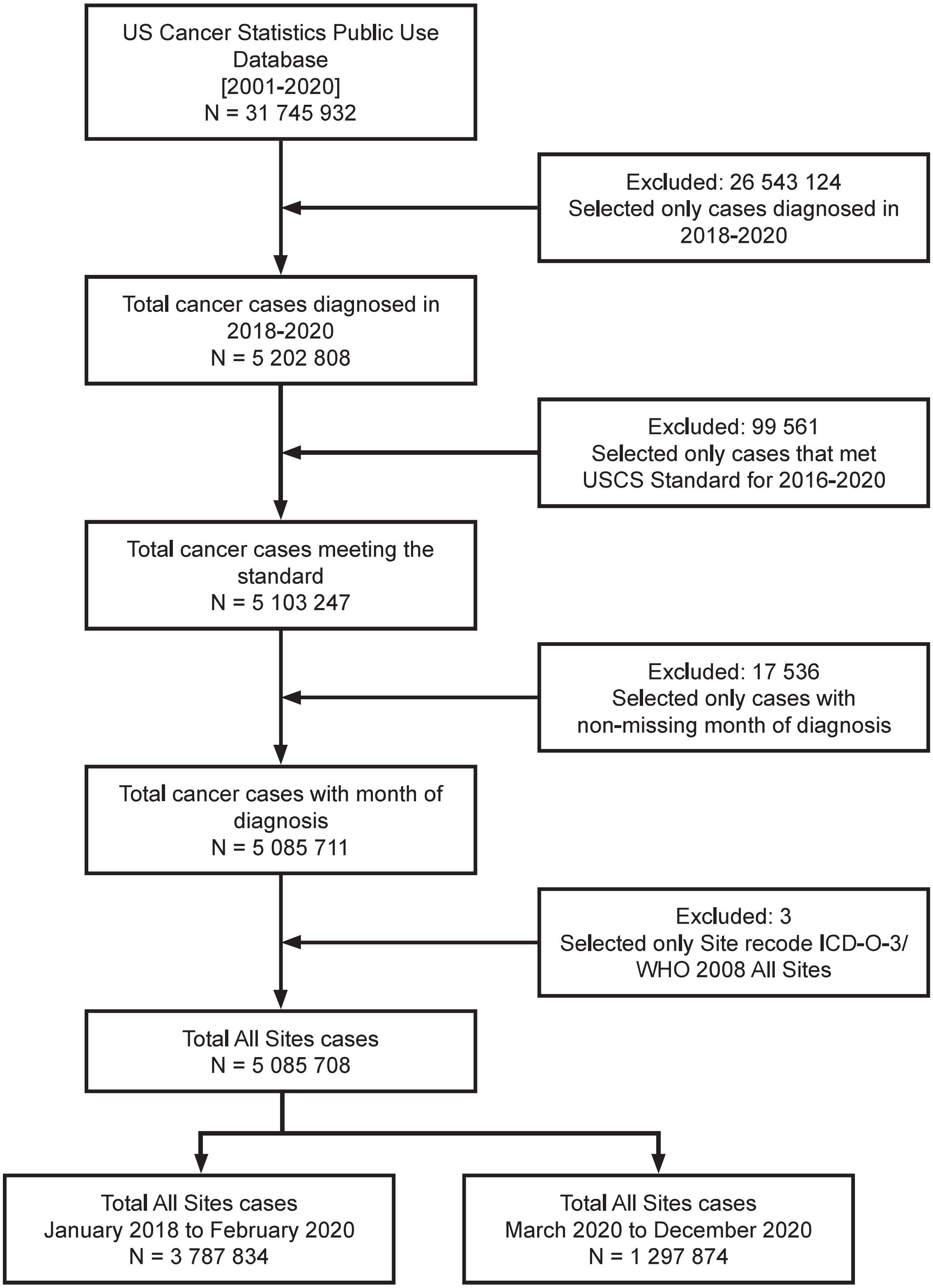

© 2024 Burus et al. JAMA Oncology.

eFigure 2. Percentage Difference in Observed from Expected Incidence Rates for Non-screenable Cancers, by Site, Stage, and Period, March-December 2020. Error bars indicate the 95% prediction interval (PI). (*) indicates significant disruption based on 95% PI not containing 0.

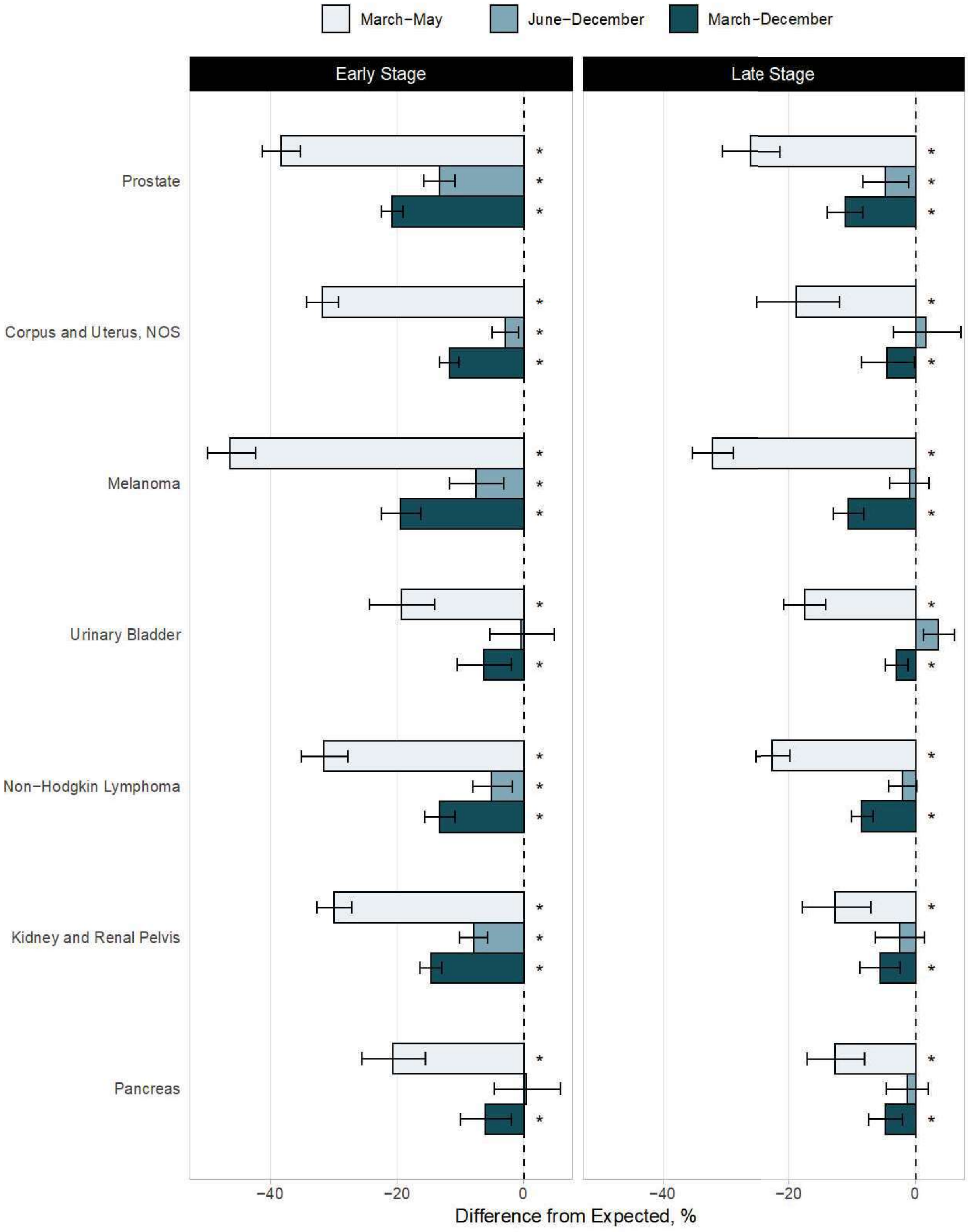

© 2024 Burus et al. JAMA Oncology.

eFigure 3. Percentage Difference in Observed from Expected All Sites Cancer Incidence Rates, by Population Subgroup and Period, March-December 2020. Error bars indicate the 95% prediction interval (PI). (*) indicates significantly worse disruption than comparison group based on non-overlapping 95% PIs.

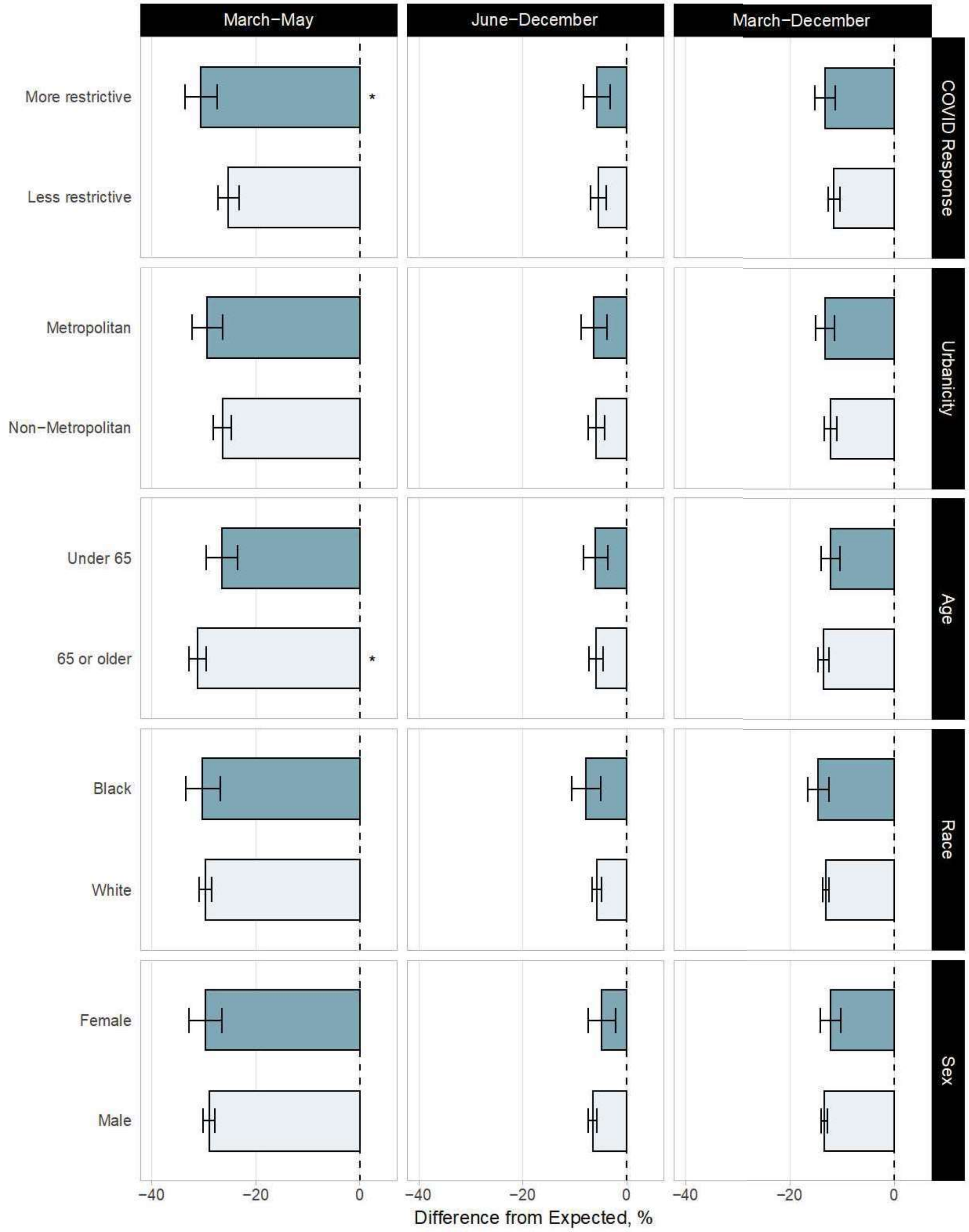

© 2024 Burus et al. JAMA Oncology.

eFigure 4. Percentage Difference in Observed from Expected Incidence Rates, by COVID Response, Site, and Period, March-December 2020. Error bars indicate the 95% prediction interval (PI). (*) indicates significantly worse disruption than comparison group based on non-overlapping 95% PIs.

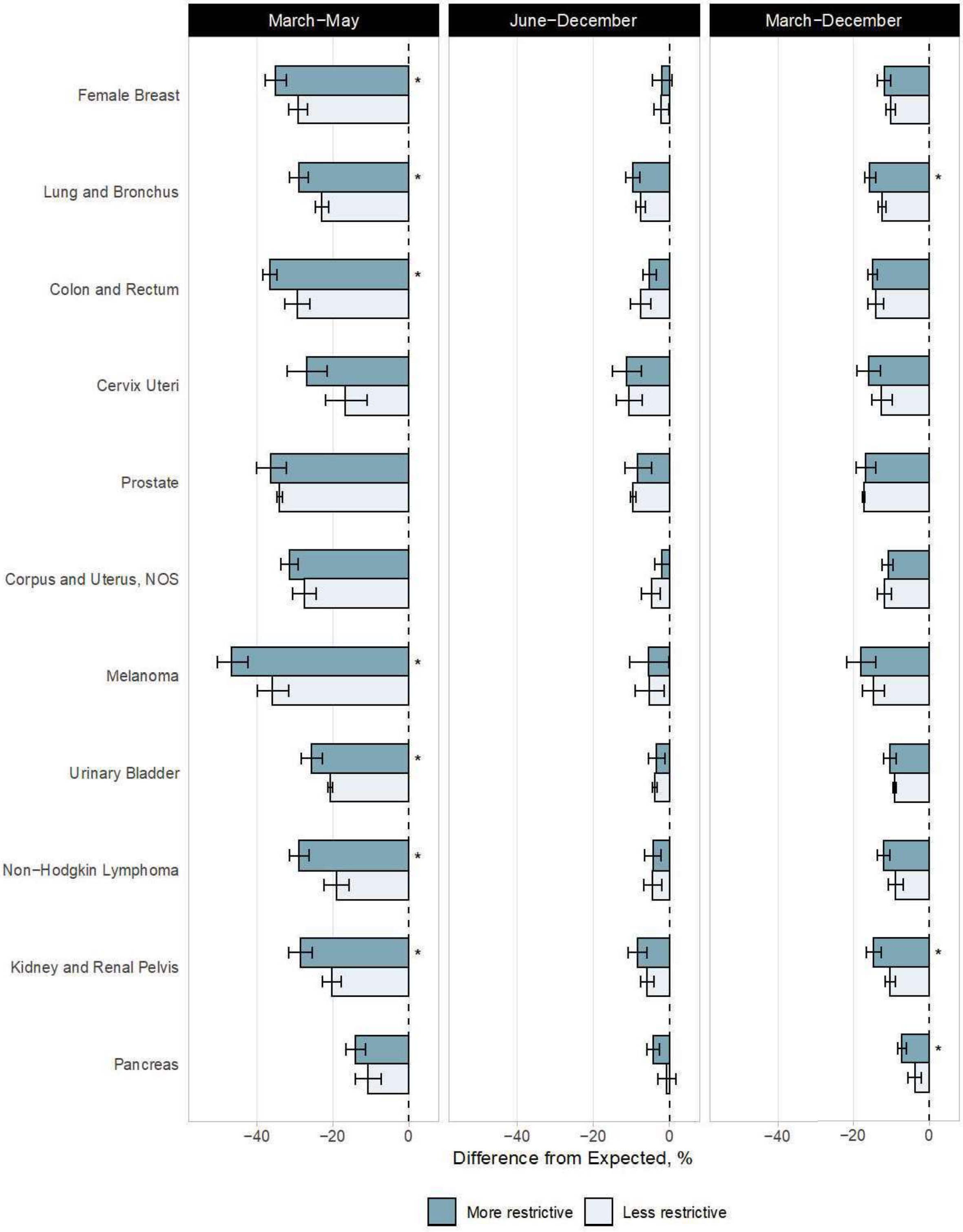

© 2024 Burus et al. JAMA Oncology.

eFigure 5. Percentage Difference in Observed from Expected Incidence Rates, by Urbanicity, Site, and Period, March-December 2020. Error bars indicate the 95% prediction interval (PI). (*) indicates significantly worse disruption than comparison group based on non-overlapping 95% PIs.

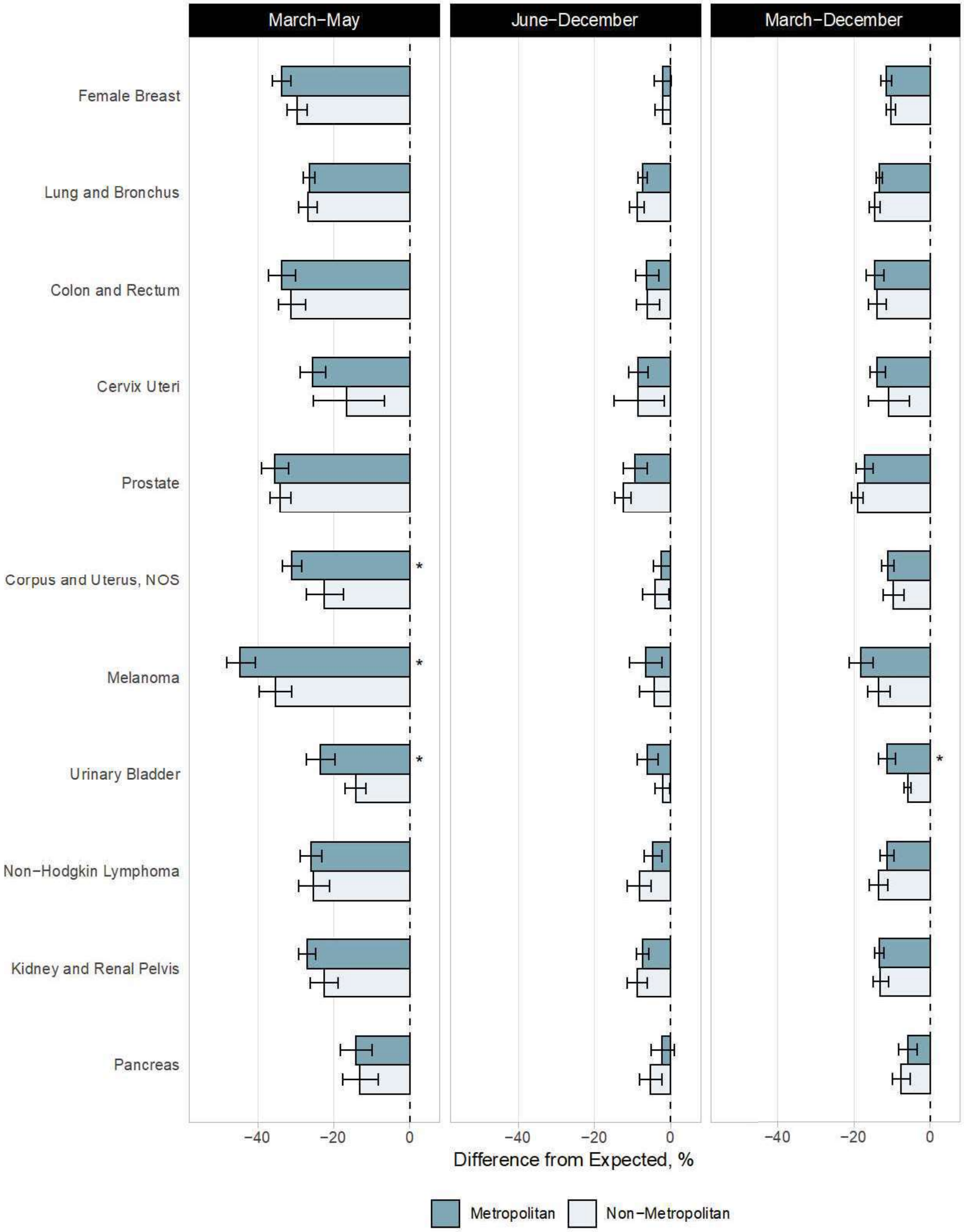

© 2024 Burus et al. JAMA Oncology.

eFigure 6. Percentage Difference in Observed from Expected Incidence Rates, by Age, Site, and Period, March-December 2020. Error bars indicate the 95% prediction interval (PI). (*) indicates significantly worse disruption than comparison group based on non-overlapping 95% PIs.

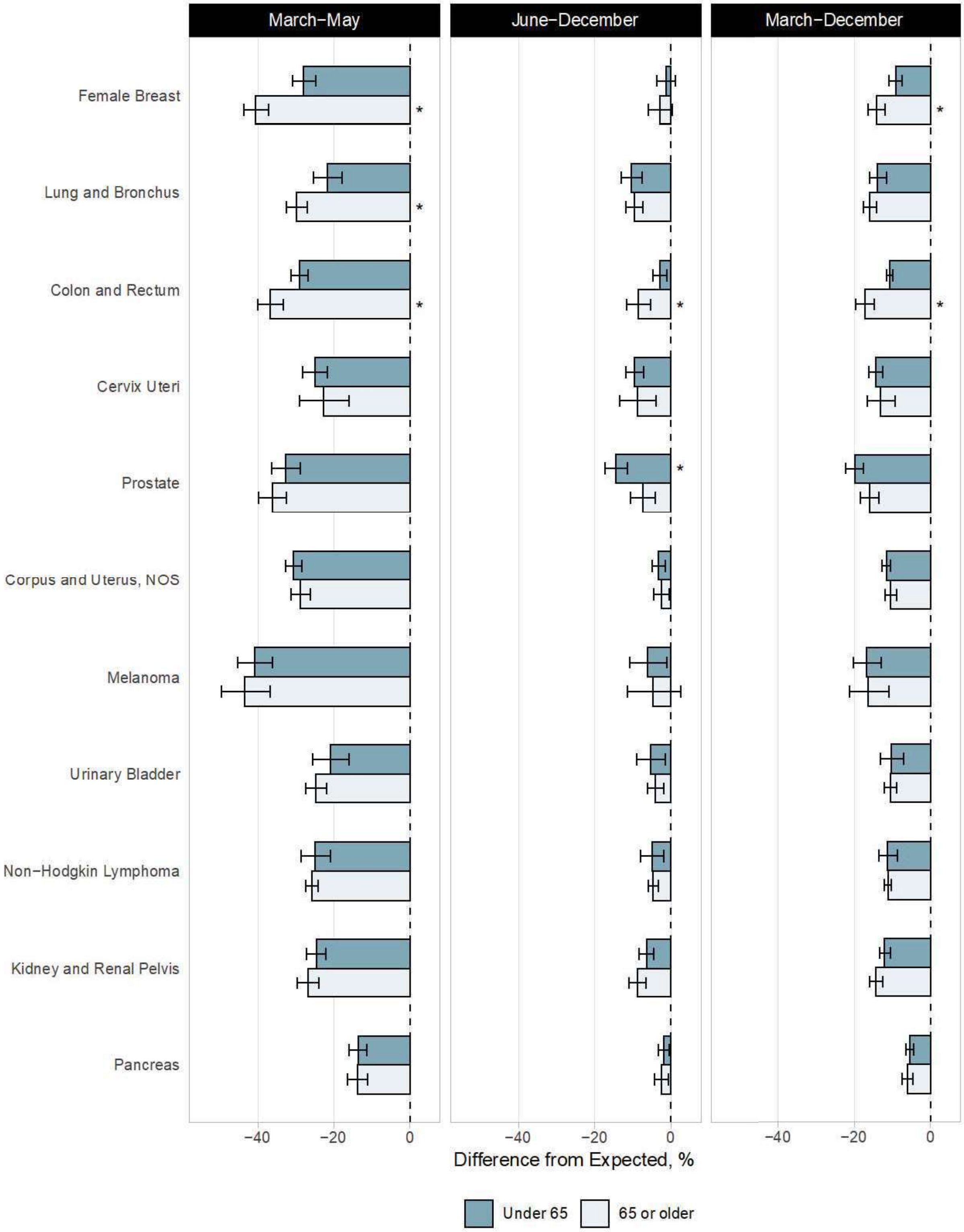

© 2024 Burus et al. JAMA Oncology.

eFigure 7. Percentage Difference in Observed from Expected Incidence Rates, by Race, Site, and Period, March-December 2020. Error bars indicate the 95% prediction interval (PI). (*) indicates significantly worse disruption than comparison group based on non-overlapping 95% PIs.

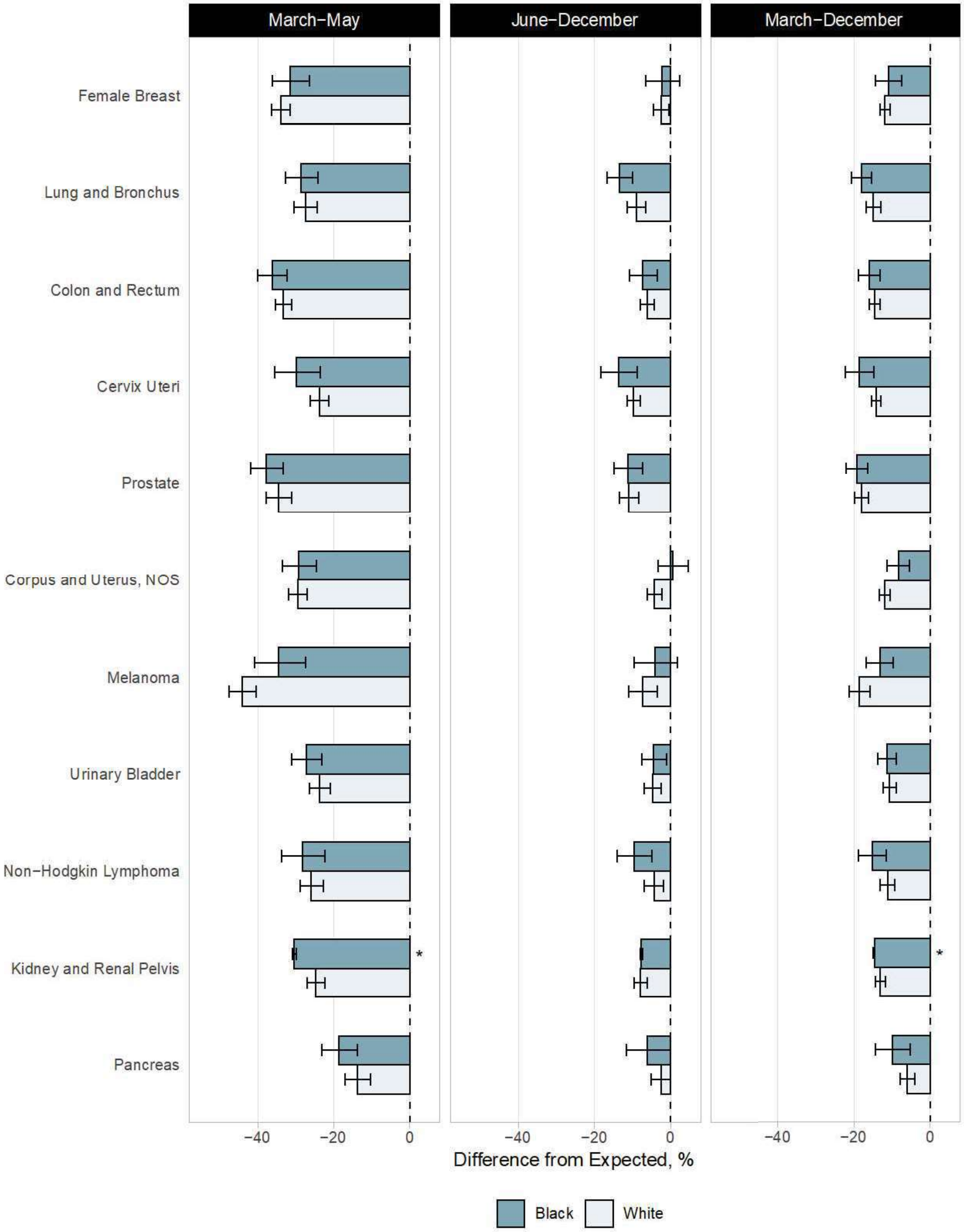

© 2024 Burus et al. JAMA Oncology.

eFigure 8. Percentage Difference in Observed from Expected Incidence Rates, by Sex, Site, and Period, March-December 2020. Error bars indicate the 95% prediction interval (PI). (*) indicates significantly worse disruption than comparison group based on non-overlapping 95% PIs.

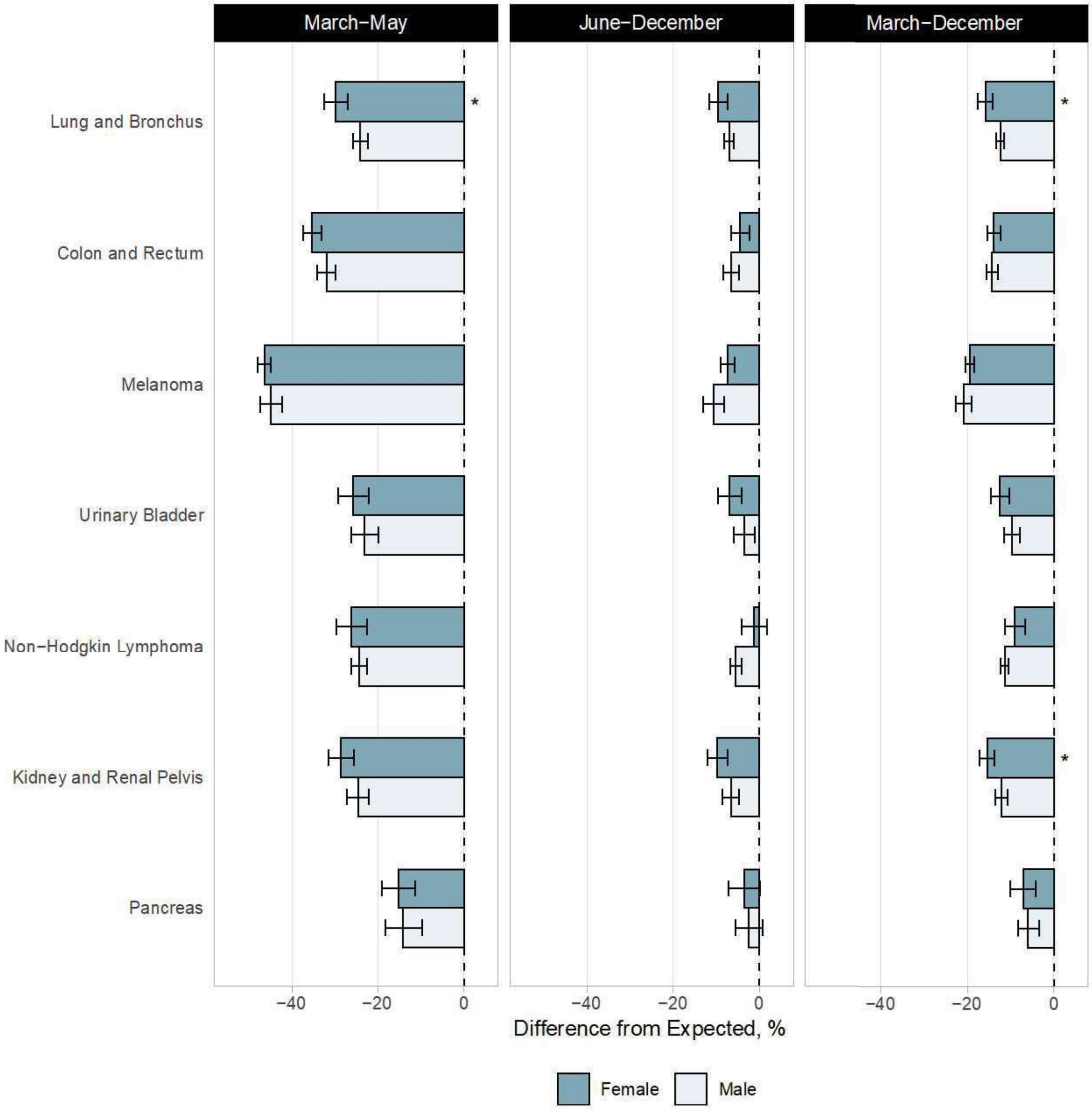

© 2024 Burus et al. JAMA Oncology.

References

1. Schaffer AL, Dobbins TA, Pearson SA. Interrupted time series analysis using autoregressive integrated moving average (ARIMA) models: a guide for evaluating large-scale health interventions. *BMC Med Res Methodol*. 2021;21(1):58. doi:10.1186/s12874-021-01235-8

2. Rob J Hyndman, George Athanasopoulos. *Forecasting: Principles and Practice*. 2nd ed. OTexts: Melbourne, Australia Accessed July 8, 2023. OTexts.com/fpp2

3. Hale T, Angrist N, Goldszmidt R, et al. A global panel database of pandemic policies (Oxford COVID-19 Government Response Tracker). *Nat Hum Behav*. 2021;5(4):529-538. doi:10.1038/s41562-021-01079-8

4. OxCGRT_USA_differentiated_withnotes_2020.csv from the Oxford Covid-19 Government Response Tracker (OxCGRT). Published online July 14, 2023.

5. National Program of Cancer Registries and Surveillance, Epidemiology, and End Results Program SEER*Stat Database: NPCR and SEER Incidence – U.S. Cancer Statistics 2001–2020 Public Use Research Database, 2022 submission (2001–2020), United States Department of Health and Human Services, Centers for Disease Control and Prevention and National Cancer Institute. Released June 2023. www.cdc.gov/cancer/uscs/public-use

© 2024 Burus et al. JAMA Oncology.
