# Supplementary material for: Undiagnosed Cancer Cases in the US During the First 10 Months of the COVID-19 Pandemic
Source: JAMA Oncol. 2024 Feb 22;10(4):500–7. doi: 10.1001/jamaoncol.2023.6969 (PMC10884945; doi:10.1001/jamaoncol.2023.6969)
Supplement: Supplement 2. — Data Sharing Statement [file jamaoncol-e236969-s002.pdf]

Data Sharing Statement

Burus. Undiagnosed Cancer Cases in the US During the First 10 Months of the COVID-19 Pandemic. *JAMA Oncol*. Published February 22, 2024. doi:10.1001/jamaoncol.2023.6969

Data
Data available: No

Additional Information
Explanation for why data not available: The data used for this study is from the U.S. Cancer Statistics (USCS) Public Use Database. Investigators can access this data through a data use agreement with USCS.
